# Deciphering hepatocellular carcinoma pathogenesis and therapeutics: a study on anoikis, ceRNA regulatory network and traditional Chinese medicine

**DOI:** 10.3389/fphar.2023.1325992

**Published:** 2024-01-12

**Authors:** Sa Guo, Nan Xing, Qinyun Du, Bin Luo, Shaohui Wang

**Affiliations:** ^1^ State Key Laboratory of Southwestern Chinese Medicine Resources, School of Pharmacy, Chengdu University of Traditional Chinese Medicine, Chengdu, China; ^2^ Shanghai Municipal Hospital of Traditional Chinese Medicine, Shanghai University of Traditional Chinese Medicine, Shanghai, China; ^3^ State Key Laboratory of Southwestern Chinese Medicine Resources, School of Ethnic Medicine, Chengdu University of Traditional Chinese Medicine, Chengdu, China; ^4^ Meishan Hospital of Chengdu University of Traditional Chinese Medicine, Meishan, China

**Keywords:** hepatocellular carcinoma, anoikis, prognostic signature, ceRNA, active compounds

## Abstract

**Introduction:** Hepatocellular carcinoma (HCC) is responsible for approximately 90% of liver malignancies and is the third most common cause of cancer-related mortality worldwide. However, the role of anoikis, a programmed cell death mechanism crucial for maintaining tissue equilibrium, is not yet fully understood in the context of HCC.

**Methods:** Our study aimed to investigate the expression of 10 anoikis-related genes (ARGs) in HCC, including BIRC5, SFN, UBE2C, SPP1, E2F1, etc., and their significance in the disease.

**Results:** Through Gene Ontology (GO) and Kyoto Encyclopedia of Genes and Genomes (KEGG) pathway analyses, we discovered that these ARGs are involved in important processes such as tissue homeostasis, ion transport, cell cycle regulation, and viral infection pathways. Furthermore, we found a significant correlation between the prognostic value of five ARGs and immune cell infiltrates. Analysis of clinical datasets revealed a strong association between BIRC5 expression and HCC pathological progression, including pathological stage, T stage, overall survival (OS), and race. By constructing a competing endogenous RNA (ceRNA) network and using molecular docking, we identified ten bioactive compounds from traditional Chinese medicine (TCM) that could potentially modulate BIRC5. Subsequent *in vitro* experiments confirmed the influence of platycodin D, one of the identified compounds, on key elements within the ceRNA network.

**Discussion:** In conclusion, our study presents a novel framework for an anoikis-centered prognostic model and an immune-involved ceRNA network in HCC, revealing potential regulatory targets. These insights contribute to our understanding of HCC pathology and may lead to improved therapeutic interventions.

## 1 Introduction

Projected forecasts indicate a concerning increase in the incidence of liver cancer, with an expected annual rise of 55.0% in new cases, potentially reaching 1.4 billion by 2040. Additionally, liver cancer deaths are projected to reach 1.3 million by that time, representing a 56.4% increase from 2020 (Rumgay et al., 2022). Hepatocellular carcinoma (HCC), which accounts for approximately 90% of these cancers, is one of the most prevalent global malignancies and the third leading cause of cancer-related deaths. The rising trend in the occurrence and mortality rates of HCC poses a significant risk to public health (Huang et al., 2022). Known factors such as smoking, infection, and alcohol consumption contribute to its development (Hashemi et al., 2023). Although standard treatments, including surgery and various therapies, often fail to improve survival outcomes due to the aggressive and drug-resistant nature of the disease, there is a crucial need to identify new prognostic markers and therapeutic targets.

Anoikis, a specialized form of cell death triggered by the detachment of cells from the extracellular matrix and neighboring cells, is increasingly acknowledged for its crucial role in various biological contexts. This includes developmental dynamics, maintenance of tissue homeostasis, disease pathogenesis, and notably, the metastatic spread of cancer cells (Han et al., 2021). Surprisingly, resistance to anoikis allows malignant cells to evade this natural death pathway, enabling their survival and establishment at distant sites in the body (Sun et al., 2022). In this context, the role of anoikis-related genes (ARGs) in cancer progression has been extensively studied. For instance, Ye et al. found that staurosporine induces resistance to anoikis and promotes metastasis in gastric cancer by up-regulating CTNNB1 and activating the Wnt/β-catenin signaling pathway (Ye et al., 2020). Other studies have also supported the importance of evading anoikis, such as the sequestration of Bim-EL in inflammatory breast cancer, as reported by (Buchheit et al., 2015; D'Amato et al., 2015). While the prognostic relevance of ARGs has been recognized in various tumor types, there is a lack of comprehensive investigation of ARGs in the context of HCC. Therefore, it is imperative for the scientific community to explore and characterize the ARGs that have prognostic significance in HCC.

The use of traditional Chinese medicine (TCM) in the management of HCC has a long historical background, spanning several centuries (Xi and Minuk, 2018). In the field of oncology, there has been increasing interest in TCM, particularly in its collection of natural compounds that may have anti-cancer properties (Wang et al., 2022). Recent studies have provided evidence of the effectiveness of compounds like shikonin, which inhibits the progression of HCC by targeting the PI3K/Akt/mTOR pathway. This leads to enhanced apoptotic and autophagic processes, which play a role in reducing the aggressiveness of cancer cells (Zhang J. et al., 2022). Other studies have highlighted the mechanism of myricetin, a compound that induces apoptosis through the mitochondrial pathway, resulting in cell cycle arrest. This apoptotic effect is attributed to the inhibition of cyclin-dependent kinase 1 (CDK1) activity, modulation of mitochondrial membrane potential, and regulation of apoptogenic factors (Zhang et al., 2011; Ji et al., 2022). These properties emphasize the potential of bioactive components in TCM formulations as promising avenues for therapeutic research in HCC.

**FIGURE 1:**
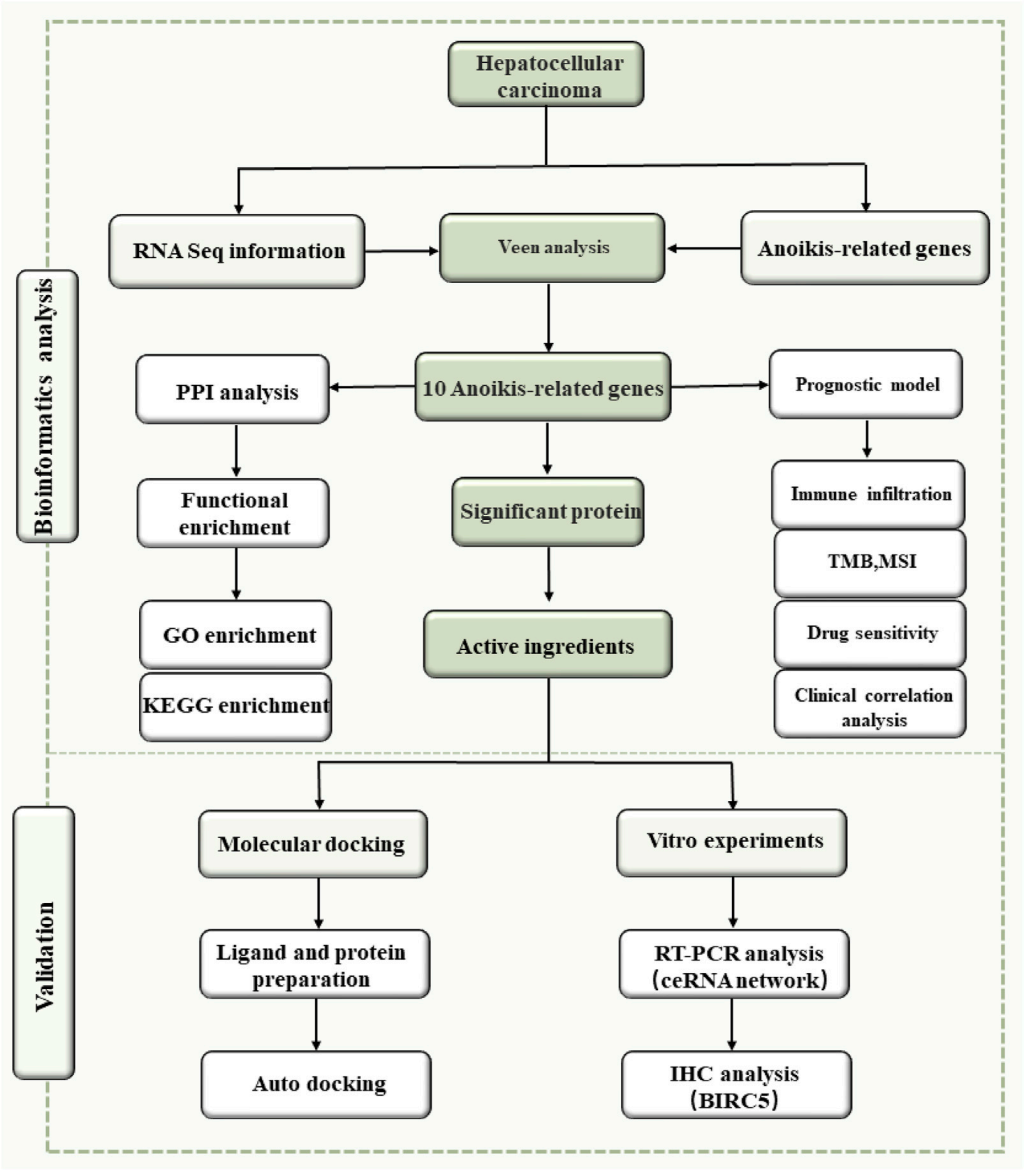
The whole flowchart of the study, showcasing the sequential workflow from data collection to analysis.

The primary objective of our investigation was to examine the prognostic significance of ARGs in HCC using a comprehensive bioinformatics approach. We aimed to elucidate the structure of competitive endogenous RNA (ceRNA) networks involving ARGs and identify specific regulatory compounds from TCM that could potentially modulate these genes. Through this research, we aim to establish a solid foundation for the development of targeted treatments for HCC that can effectively target anoikis mechanisms. The step-by-step synthesis of our research methodology is systematically illustrated in Figure 1.

## 2 Materials and methods

Cell lines LX2, HepG2, and Huh7 were obtained from the Cell Bank of the Chinese Academy of Sciences. Baicalin, platycodin D, and resveratrol were obtained from Chengdu Lemeitian Pharmaceutical Technology Co., Ltd. (Chengdu, China). Reagents for nucleic acid extraction and cDNA synthesis, including TransZol Up Plus RNA kits and First-Strand cDNA Synthesis Kits, were provided by Quanshi Jin Biotechnology Co., Ltd. (Nanjing, China). MagicSYBR Mixture for quantitative PCR was supplied by Jiangsu Cowin Biotech Co., Ltd. (Taizhou, China). MiRNA extraction and amplification kits, namely, MiPure Cell/Tissue miRNA Kit, miRNA 1st Strand cDNA Synthesis Kit, and miRNA universal SYBR qPCR Master Mix, were provided by Novozan Biotechnology Co., Ltd. (Nanjing, China). Cell viability assays were conducted using CCK8 assay kits from Jiangsu Baoguang Biotechnology Co., Ltd. (Wuxi, China). Cell culture essentials such as Dulbecco’s modified eagle medium (DMEM), Phosphate buffer solution (PBS), Trypsin digestive solution, and Penicillin-streptomycin solution were obtained from Invitrogen Life Technology Co., Ltd. (California, US). For immunodetection, BIRC5 (TA6017) antibody was procured from Abmart Shanghai Co., Ltd. (Shanghai, China), and Alexa Fluor 488 secondary antibody from Cell Signaling Technology (Boston, US).

### 2.1 Data preparation

RNA sequencing data relevant to HCC were obtained from the University of California Santa Cruz (UCSC) Xena database (https://xena.ucsc.edu/). The dataset consisted of 423 profiles, with 50 samples designated as normal hepatic tissue and 373 samples representing HCC specimens. Our analysis focused on identifying differentially expressed genes within this cohort. We applied stringent screening criteria, using a Log2-fold change (FC) threshold of ≥2 and a significance cut-off of *p* < 0.05. To identify ARGs (Aberrantly Expressed Genes), we utilized the GeneCards repository (https://www.genecards.org/), which provided a list of 455 ARGs with a relevance score exceeding 2. By employing Venn diagram analysis tools (https://bioinformatics.psb.ugent.be/webtools/Veen/), we identified 10 significant ARGs. For obtaining associated clinical information, we utilized resources from the USCA database.

### 2.2 Enrichment analysis

To investigate the potential biological functions and underlying mechanisms of the 10 identified ARGs, we performed Gene Ontology (GO) and Kyoto Encyclopedia of Genes and Genomes (KEGG) pathway enrichment analyses. These analyses were conducted using the Metascape database (https://metascape.org/), a comprehensive suite designed to decipher gene functionalities and provide context to gene clusters. The enriched functional categories and pathways identified through these analyses were visually represented using the “clusterProfiler” package in the R programming environment. This package is a powerful tool for statistical evaluation and graphical representation, allowing for a detailed comparison of biological themes among gene sets.

### 2.3 Establishment of ARGs prognostic signature model

To evaluate the prognostic value of the 10 identified ARGs for overall survival (OS) in HCC, we utilized R software packages “survminer” and “survival”, aligning our methods with the model employed in the study by (Wang et al., 2022). To refine our prognostic assessment, we implemented the Least Absolute Shrinkage and Selection Operator (LASSO) Cox regression analysis, a technique well-suited for reducing high-dimensional data and developing prognostic models. The resulting prognostic model categorized patients into low-risk and high-risk subgroups, and their OS disparities were evaluated through Kaplan-Meier survival analysis. Subsequently, we assessed the reliability of the ARG-derived risk scores using receiver operating characteristic (ROC) analysis. The ROC analysis helps determine the predictive accuracy of the risk scores in forecasting patient outcomes. These statistical analyses, performed using R software, established that a *p* < 0.05 would indicate statistical significance. The integration of these methodologies allowed for a comprehensive assessment of the prognostic implications of ARGs in HCC.

### 2.4 Analysis of immune infiltration, tumor mutation burden, microsatellite instability, and drug sensitivity

To investigate the association between the expression of ARGs and immune cell infiltration in the tumor microenvironment, we utilized single-sample Gene Set Enrichment Analysis (ssGSEA). This method quantitatively evaluated the presence and abundance of 24 different immune cell types. To understand the correlation between the expression of 5 important ARGs and key immunogenomic markers (Tumor Mutation Burden and Microsatellite Instability), we conducted Spearman correlation analysis. A significance level of *p* < 0.05 was used to determine statistical significance. Additionally, we examined the sensitivity of ARGs to various chemotherapeutic agents using the Genomic Cancer Drug Sensitivity in Cancer (GSCA) database (http://bioinfo.life.hust.edu.cn/GSCA/#/). This database provides access to a wide range of pharmacogenomic datasets, allowing for the identification of potential drug targets in different cancer subtypes. By employing these analytical approaches, we were able to systematically explore the relationship between ARGs, immune activity, and drug responsiveness in cancer.

### 2.5 Protein expression evaluation and establishment of ceRNA regulatory network

To visualize the protein expression patterns of BIRC5 and SPP1, we accessed the Human Protein Atlas (HPA, https://www.proteinatlas.org/). This allowed us to compare the levels of these proteins in normal liver tissue and HCC. In addition, we used the StarBase platform (https://starbase.sysu.edu.cn/) to predict potential miRNA targets of BIRC5 and SPP1. We employed the Mann-Whitney *U* test and Kaplan-Meier mean analysis to examine the expression dynamics and prognostic relevance of miRNAs associated with BIRC5 and SPP1 in HCC. Our analysis focused on miRNAs that showed statistically significant differences. To further explore the role of long non-coding RNAs (lncRNAs) associated with these miRNAs, we utilized the LncBase and StarBase databases. We evaluated the expression patterns and prognostic implications of these lncRNAs using the HCC dataset from The Cancer Genome Atlas (TCGA), considering a *p* < 0.05 as statistically significant. Ultimately, we established a ceRNA regulatory network centered on BIRC5, which provides new insights into the molecular interactions and clinical significance in HCC.

### 2.6 Molecular docking

The three-dimensional (3D) structural configuration of the bioactive compound analyzed in our study was obtained from the Traditional Chinese Medicine Systems Pharmacology Database and Analysis Platform (TCMSP, https://old.tcmsp-e.com/). The protein data bank (PDB) file for the target protein BIRC5 was sourced from the RCSB Protein Data Bank (HYPERLINK "https://www.rcsb.org" \o "https://www.rcsb.org"https://www.rcsb.org). Using these structural data, we conducted molecular docking analysis using Autodock Vina software to investigate the docking interactions between the selected traditional Chinese medicine active ingredient and the BIRC5 protein. The *in silico* binding affinities provided valuable insights into potential interactions that may contribute to therapeutic efficacy.

### 2.7 Quantitative real time polymerase chain reaction

Total RNA extraction was performed using the TransZol Up Plus RNA Kit to isolate high-quality RNA samples. After extraction, the RNA was quantified and its purity was assessed using the Nanodrop Micro Nucleic Acid Analyzer (Thermo Fisher Scientific, USA). The expression levels of the target genes were then measured quantitatively using the Quantitative Real-Time Fluorescence PCR Detection System (Rocgene, Beijing, China). The primer sequences used for amplification are listed in Table 1. To determine the relative mRNA expression of the genes of interest, the 2^−Δ(ΔCt)^ method was calculated, where Δ(ΔCt) represents the difference in cycle threshold values normalized to a reference gene.

**TABLE 1:** qRT-PCR primer sequences.

Genes	Primer	Sequences 5′–3′
BIRC5	F	TTG GCC CAG TGT TTC TTC TGC TT
	R	GCA CTT TCT CCG CAG TTT CCT CA
hsa-miRNA-204-5p	sl	GTC GTA TCC AGT GCA GGG TCC GAG GTA
	sl	TTC GCA CTG GAT ACG ACA GGC AT
	F	CGC GTT CCC TTT GTC ATC CT
	R	AGT GCA GGG TCC GAG GTA TT
U6	F	CTC GCT TCG GCA GCA CA
	R	AAC GCT TCA CGA ATT TGC GT
OIP5-AS1	F	GCT TCC TTC CTT TCC CTT GCT CA
	R	TGC ACT AAC CCC TAA CAT GGC AC
DCP1A	F	CTG GAT TGT TGG CCC TCT GAC TC
	R	CTG GAA GGT CAAGGCTGCATGAG
PPP1R9B	F	GAA CAG CTC CAA CCT CTC CAC AC
	R	TTG GAG AGA GAC AAC AGA GGG GT
GAPDH	F	TGA CTT CAA CAG CGA CAC CCA
	R	CAC CCT GTT GCT GTA GCC AAA

### 2.8 Immunofluorescence staining

HepG2 and Huh7 cells were cultured in 6-well plates with cell crawlers. The plates were then placed in a cell incubator for 24 h to ensure adherence and growth. After incubation, the cells were treated with various protocols, including fixation, permeabilization, and blocking. These steps aimed to preserve cellular structures, allow the ingress of antibodies, and prevent non-specific binding. Next, the cells were incubated overnight at 4°C with a primary antibody specific for BIRC5, enabling primary immune-detection. The following day, the cells were incubated at room temperature for 1 h with the fluorescently-labeled Alexa Fluor 488 secondary antibody, facilitating secondary immune-detection. To complete the staining procedure, the cell nuclei were counterstained with DAPI for 5 min, resulting in a distinct blue fluorescence. The cells were then mounted in glycerol to preserve the fluorescence for imaging. Immunofluorescence imaging was performed using an Olympus fluorescence microscope. These images visually depict the presence and localization of the BIRC5 protein within the cells, providing insights into its expression and potential functional implications in the studied cell lines.

### 2.9 Statistical analysis

Data analysis and visualization were performed using the R package and GraphPad Prism 7.0, respectively. The data are presented as the mean ± standard error of the mean (SEM) from three independent experiments.

## 3 Results

### 3.1 ARGs expression, PPI, and gene mutation in HCC

**FIGURE 2:**
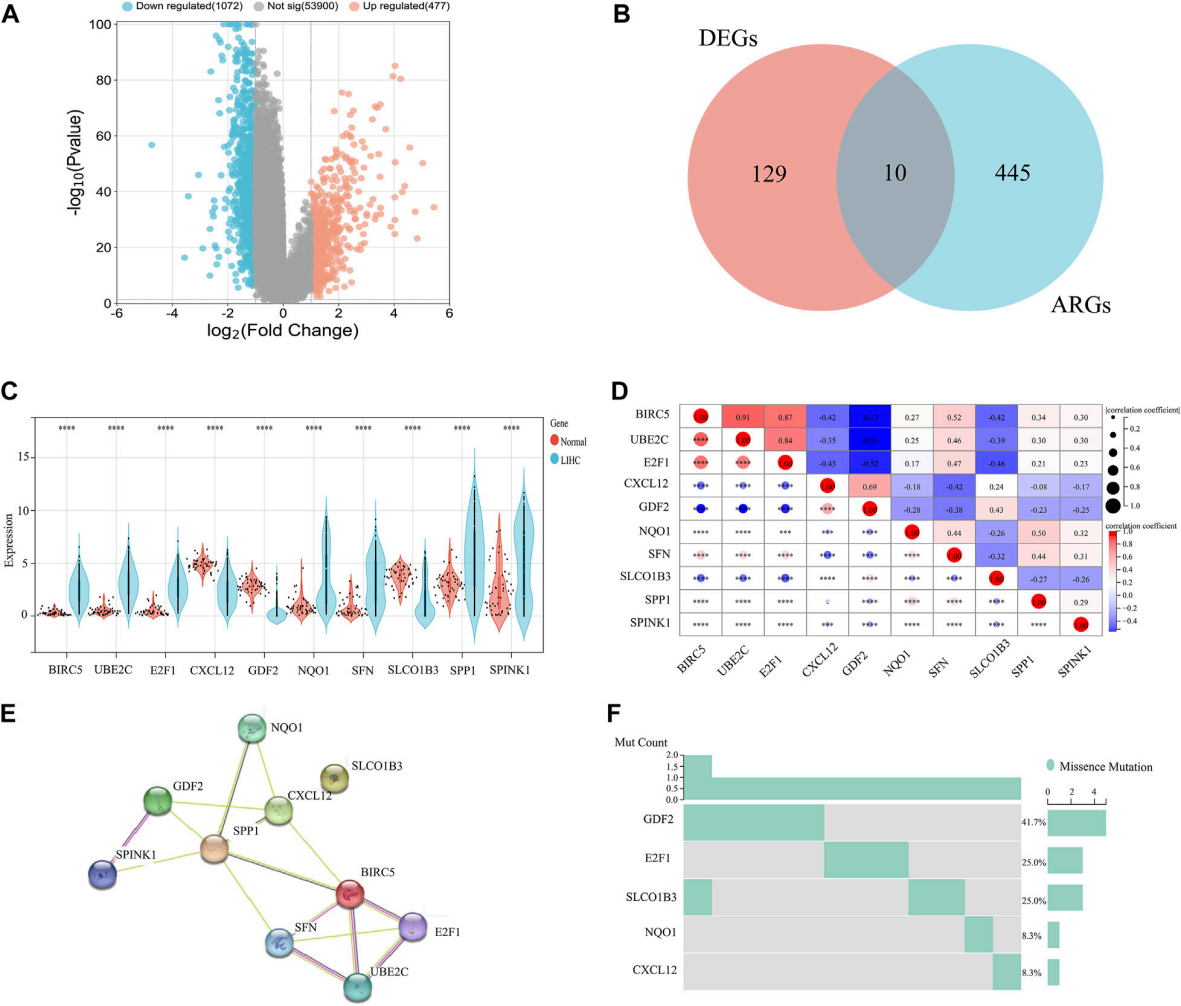
Comparative analysis of ARG expression levels and mutational status in HCC *versus* normal tissue samples. **(A)** Volcanic map. **(B)** Venn diagram. **(C)** Differential expression analysis. **(D)** Correlation analysis. **(E)** PPI network analysis. **(F)** Mutation status analysis of HCC versus normal tissue samples.

Our investigation utilized Venn diagram analysis to identify a group of 10 crucial genes related to ARGs, as shown in Figure 2A. After analyzing RNA sequences, we confirmed significant differences in the transcriptional profiles of these ARGs compared to similar sequences from a control group, with a *p*-value of less than 0.0001 (Figure 2B). Notably, we found increased expression of several genes, including Baculoviral IAP repeat-containing 5 (BIRC5), Ubiquitin-conjugating enzyme E2C (UBE2C), E2F transcription factor 1 (E2F1), NADPH: quinone oxidoreductase1 (NQO1), Single frequency network (SFN), Secreted phosphoprotein 1 (SPP1), and serine protease inhibitor kazal type 1 (SPINK1). Additionally, we observed a decrease in the expression of Chemokine (C-X-C motif) ligand 12 (CXCL12), growth differentiation factor 2 (GDF2), and solute carrier organic anion transporter family member 1B3 (SLCO1B3).

A heat map analysis revealed strong correlations between genes (*p* < 0.0001) (Figure 2C), suggesting coordinated regulation within the ARG group. To further understand these connections, we constructed a protein-protein interaction (PPI) network based on the identified ARGs (Figure 2D). Analysis of the network revealed significant links among BIRC5, UBE2C, E2F1, CXCL12, GDF2, NQO1, SFN, SPP1, and SPINK1, supporting the associations observed in the heat map analysis (Figure 2E). Additionally, we performed mutation profiling of the 10 ARGs in HCC specimens. This analysis identified variations primarily in GDF2, E2F1, SLCO1B3, NQO1, and CXCL12. Notably, GDF2 had the highest mutation incidence at 41.7%, while E2F1 and SLCO1B3 each had mutations in 25% of the cases (Figure 2F). These findings provide important genetic information that may contribute to our understanding of the mechanisms and potential therapeutic targets in HCC.

### 3.2 Enrichment analysis

**FIGURE 3:**
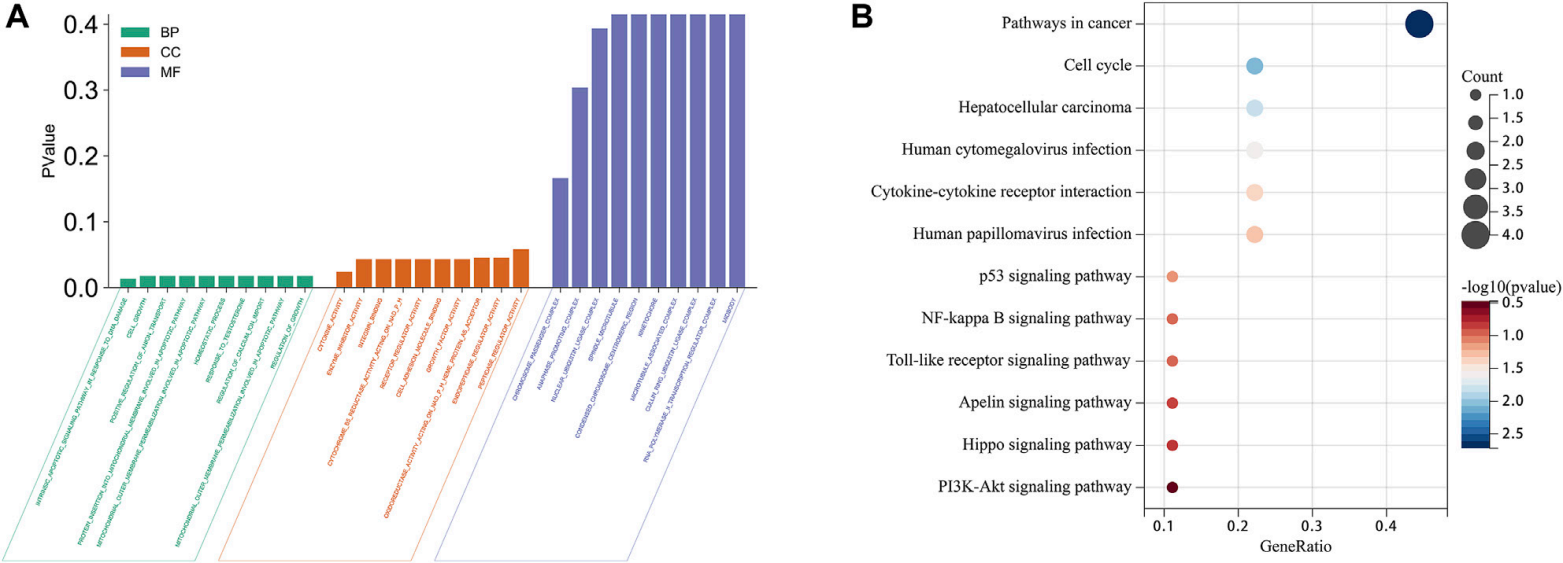
GO and KEGG enrichment analyses of 10 ARGs in HCC. **(A)** Bubble plot of GO enrichment results of 10 ARGs in HCC. **(B)** Bubble plot of KEGG enrichment results of ARGs in HCC.

To investigate the mechanisms of action of the 10 identified ARGs in HCC, we conducted GO and KEGG enrichment analyses. These analyses provided insights into the potential biological roles and molecular mechanisms of the ARGs. The GO functional analysis revealed a significant enrichment of the ARGs in various biological processes (BPs) and molecular functions (MFs), as shown in Figure 3A. Enriched BPs included regulation of homeostatic processes, ion transport, and cell differentiation. In terms of molecular functions, the ARGs were found to be enriched in enzyme binding, cytokine activity, and enzyme inhibitor activity. Additionally, the cellular components (CCs) analysis indicated that these genes are localized in structural entities such as the microtubule cytoskeleton, mitochondria, and chromosomes.

Complementing these insights, the KEGG pathway analysis revealed that the ARGs were associated with various pathways that are significant in cancer biology and HCC pathogenesis. These pathways include those involved in cancer proliferation and progression, the cell cycle, and specifically related to HCC. Furthermore, the ARGs played a prominent role in infection-related pathways, such as those triggered by human cytomegalovirus infection, as well as in signaling cascades crucial for cell communication and survival. These signaling cascades include cytokine-cytokine receptor interaction, the Phosphoinositide 3-kinase (PI3K)/Protein Kinase B (Akt) signaling pathway, Toll-like receptor (TLR) signaling pathway, and the p53 signaling pathway, as shown in Figure 3B. Collectively, these results provide valuable insights into the diverse roles that ARGs may play in the context of HCC, laying the groundwork for the development of targeted therapeutic strategies.

### 3.3 Establishment of ARGs prognostic signature model

**FIGURE 4:**
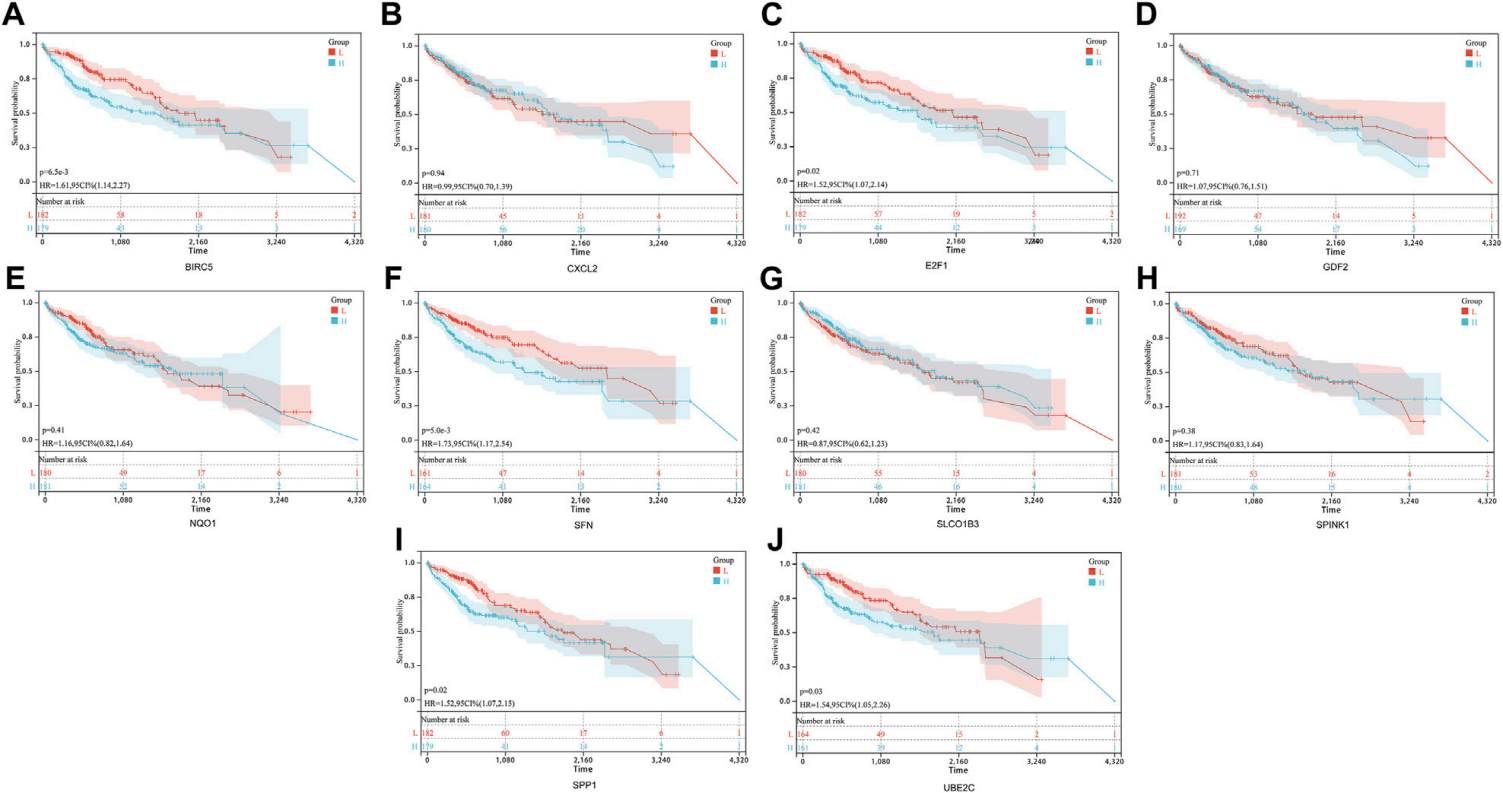
Prognostic value of 10 ARGs in HCC. The OS curves of BIRC5 **(A)**, CXCL2 **(B)**, E2F1 **(C)**, GDF2 **(D)**, NQO1 **(E)**, SFN **(F)**, SLCO1B3 **(G)**, SPIN **(H)**, SPP1 **(I)**, and UBE2C **(J)** in patients with HCC in the low and high expression groups.

**FIGURE 5:**
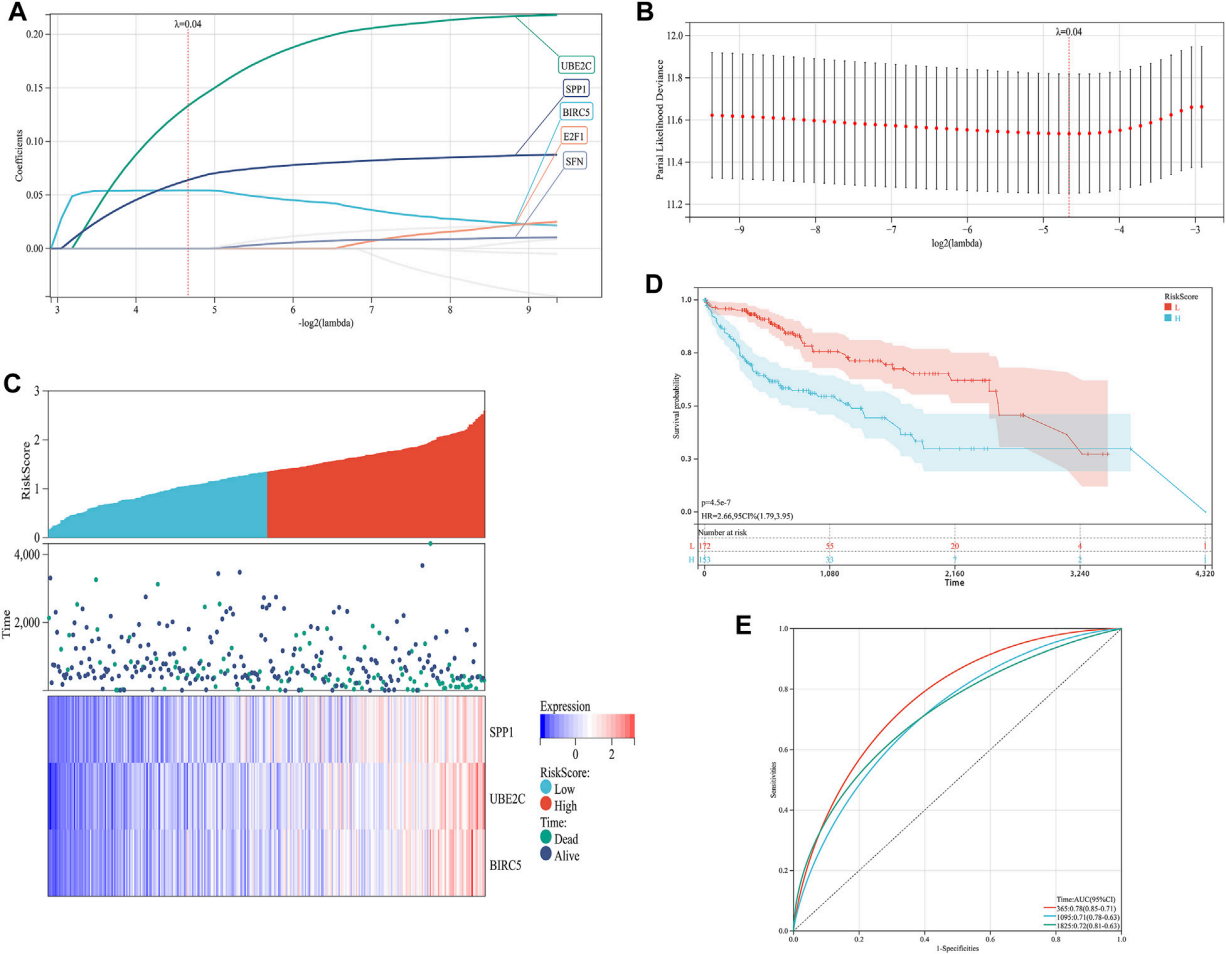
Construction of a prognostic signature model of CRGs in HCC. **(A)** LASSO coefficients of the five prognostic ARGs. **(B)** PLD of the five prognostic ARGs. **(C)** Distribution of risk score, survival status, and expression of the five prognostic ARGs. **(D)** OS curve of patients with HCC in the low and high expression groups. **(E)** 1-, 3-, and 5-year ROC prediction curves for patients with HCC.

In order to establish a prognostic signature model informed by the involvement of ARGs in HCC, we conducted a univariate Cox regression analysis. This initial assessment revealed that five out of the ten ARGs (specifically BIRC5, E2F1, SFN, SPP1, and UBE2C) showed a significant correlation with patient outcomes, as depicted by individual Kaplan-Meier curves (Figures 4A–J). To improve the precision of the prognostic signature model, we utilized the LASSO Cox regression methodology, which incorporated these five ARGs with substantial prognostic value. Risk scores were calculated using a formula based on the work of Wang et al. (Wang et al., 2022) and the coefficients of the prognostic signature coupled to the partial likelihood deviance for HCC (Figures 5A, B). Co-expression analysis of these five ARGs revealed a strong inverse relationship with the occurrence of HCC, particularly for BIRC5, SPP1, and UBE2C (Figure 5C). The overall survival (OS) curve showed a clear trend, with patients classified in the high-risk group based on the risk scores experiencing increased mortality risk and shorter survival times (*p* = 4.5e-7, HR = 2.66) (Figure 5D). The prognostic validity of the model was further supported by ROC curve analysis, which yielded area under the curve (AUC) values of 0.78, 0.71, and 0.72 for the 1, 3, and 5-year benchmarks, respectively, confirming the model’s effectiveness in predicting long-term survival (Figure 5E). These comprehensive analyses demonstrate the potential of the ARG-based prognostic model in forecasting clinical outcomes for HCC patients.

### 3.4 Immune infiltration analysis of critical ARGs

**FIGURE 6:**
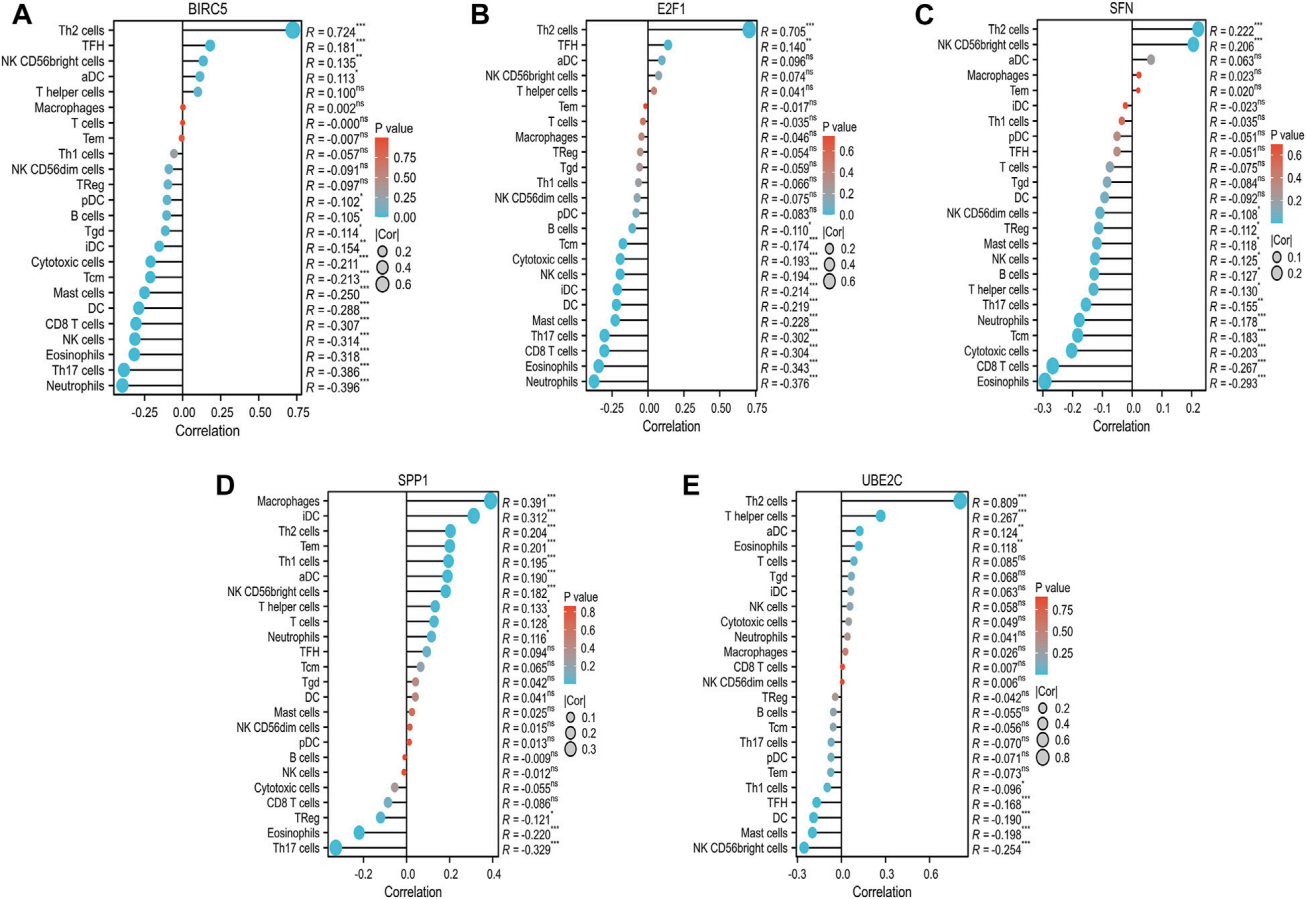
Correlation between the five prognostic ARGs and immune infiltration in HCC. The correlation between BIRC5 **(A)**, E2F1 **(B)**, SFN **(C)**, SPP1 **(D)**, UBE2C **(E)**, and the degree of immune infiltration of 24 immune cell types in patients with HCC.

**FIGURE 7:**
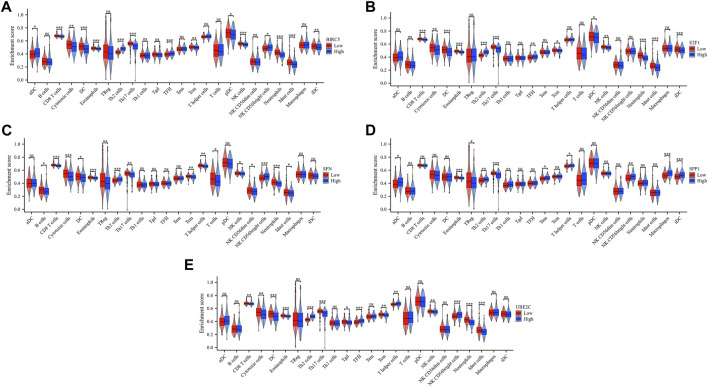
Enrichment scores of the five prognostic ARGs in 24 immune cell types in HCC. The five prognostic ARGs were BIRC5 **(A)**, E2F1 **(B)**, SFN **(C)**, SPP1 **(D)** and UBE2C **(E)**.

To enhance our understanding of the immunogenic context in which a group of critical ARGs (BIRC5, E2F1, SFN, SPP1, and UBE2C) operate, we investigated their potential associations with various immune cell subsets, encompassing 24 distinct types. We found that these ARGs showed significant correlations with the distribution and composition of immune cells, including Th2 cells, mast cells, CD8 T cells, natural killer (NK) cells, eosinophils, Th17 cells, and neutrophils. Specifically, BIRC5, E2F1, and SFN were positively associated with Th2 cells, while showing an inverse relationship with immune cell types that are important for antitumor immunity, such as neutrophils, eosinophils, CD8 T cells, Th17 cells, mast cells, and NK cells (Figures 6A–C). Interestingly, SPP1 was positively associated with Th2 cells and neutrophils, but inversely correlated with Th17 cells and eosinophils (Figure 6D). UBE2C showed a favorable association with Th2 cells and eosinophils, but was inversely associated with mast cells (Figure 6E). Further analysis using ssGSEA confirmed these findings, with all ARGs showing significant enrichment across diverse immune cell types. The enrichment scores were 15, 13, 11, 6, and 12 for BIRC5, E2F1, SFN, SPP1, and UBE2C, respectively (Figures 7A–E). These results highlight the strong association of these prognostically relevant ARGs with the immune cell infiltration observed in the HCC landscape, suggesting their potential role in the immune response.

### 3.5 TMB, MSI, and drug sensitivity analyzes

**FIGURE 8:**
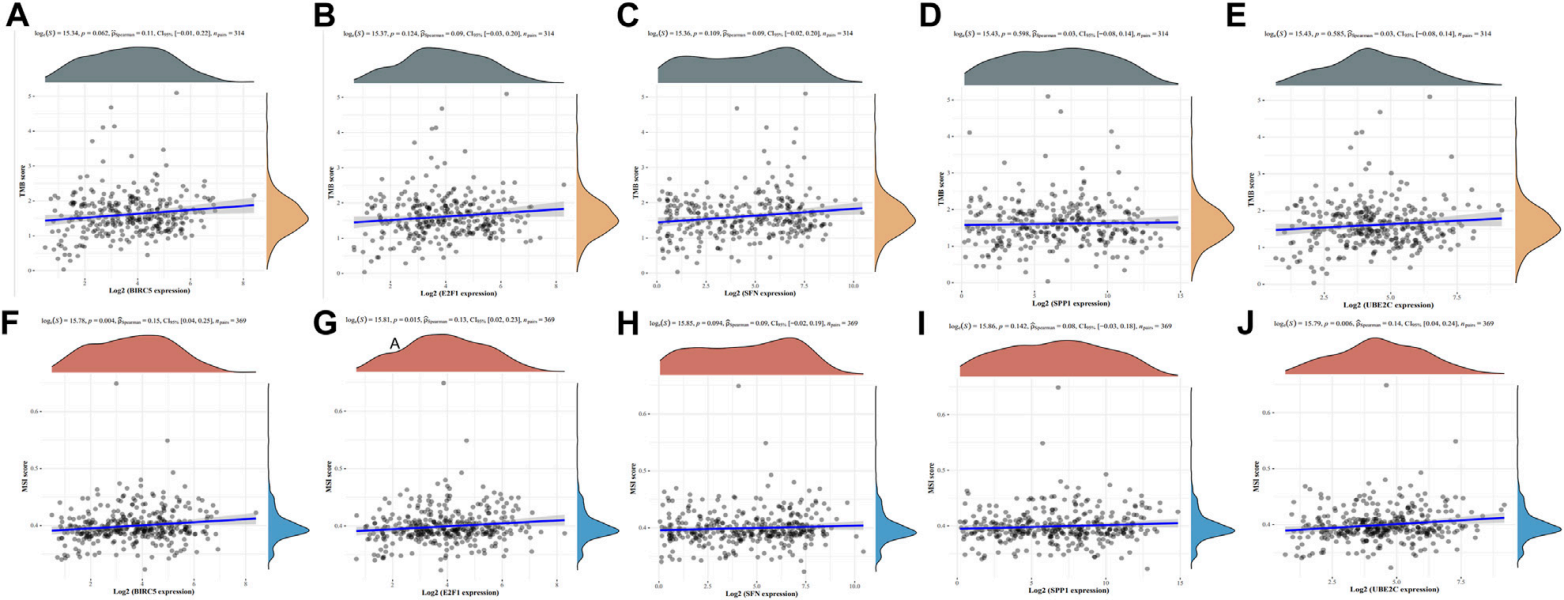
Correlation analysis of the five ARGs with TMB and MSI in HCC. **(A–E)** Correlation between the five ARGs and TMB in LUAD. **(F–J)** Correlation between the five ARGs and MSI in HCC.

To investigate the potential of the five ARGs as biomarkers for guiding drug discovery efforts in HCC, we examined their relationships with TMB and MSI. TMB and MSI are important genomic signatures that are often considered in cancer prognosis, therapy prediction, and immunotherapy responsiveness. Our analysis revealed that while BIRC5 (Figure 8A), E2F1 (Figure 8B), SFN (Figure 8C), SPP1 (Figure 8D), and UBE2C (Figure 8E) did not show statistically significant correlations with TMB, interesting associations were observed between MSI and the expression of certain ARGs. Specifically, BIRC5 (Figure 8A), E2F1 (Figure 8B), and UBE2C (Figure 8E) exhibited a positive association with MSI. These findings suggest a potential link between MSI status and the expression of specific ARGs in HCC, which could provide insights into tumor behavior and potential sensitivities to anti-cancer drugs.

The relationship between drug susceptibilities, as indicated by the CTRP and GDSC databases, and the mRNA expression levels of the five ARGs was examined. Analysis of data from the Genomics of Drug Sensitivity in GSCA revealed an inverse correlation between the mRNA expression levels of BIRC5 and E2F1 and the effectiveness of several drugs. On the other hand, SFN and SPP1 showed a positive correlation, as depicted in Supplementary Figure S1. These findings lay the groundwork for further exploration of the potential use of these ARGs as biomarkers for drug response in HCC. Additionally, they may drive the development of new therapeutic strategies tailored to the molecular characteristics of individual tumors.

### 3.6 Clinical correlation analysis

**FIGURE 9:**
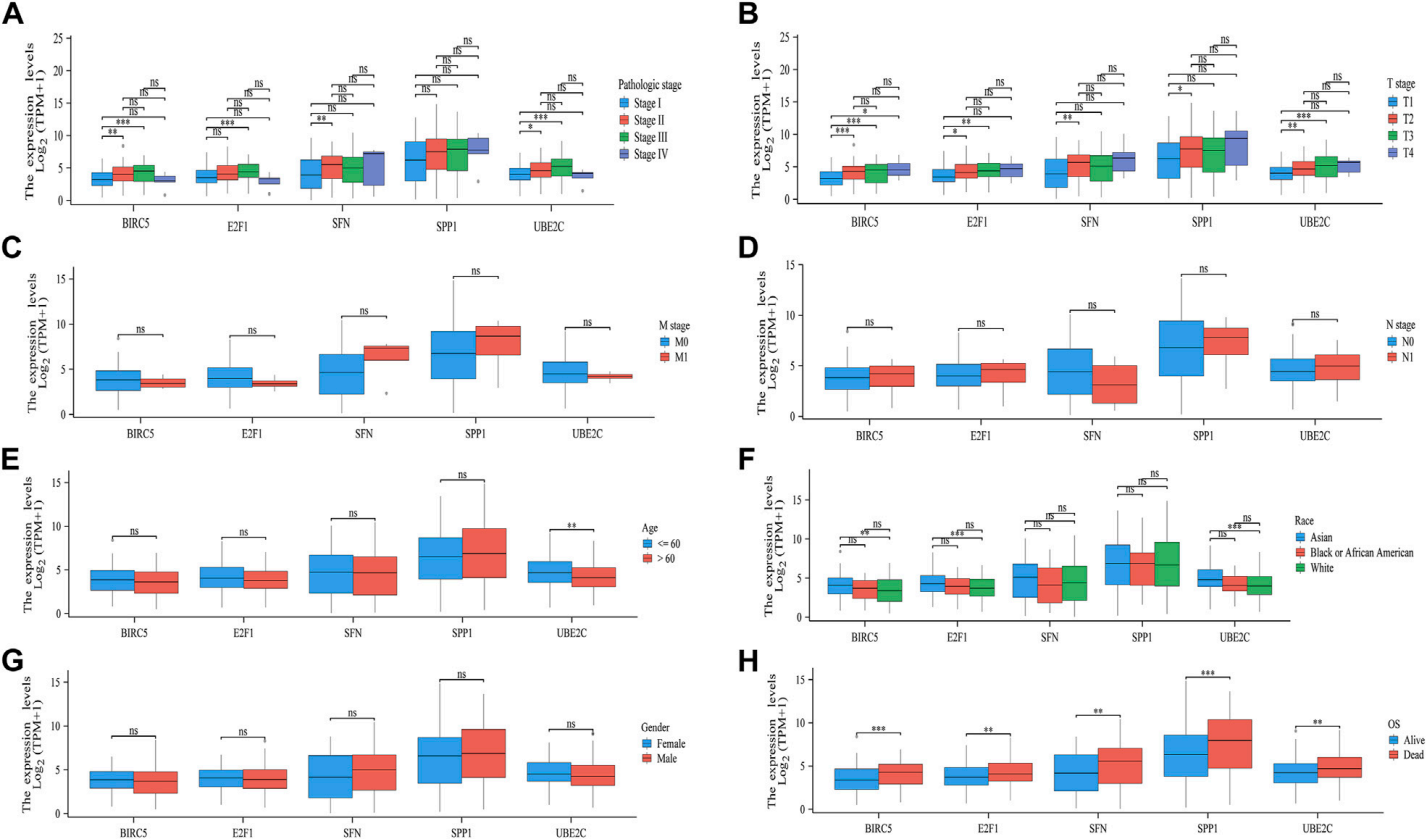
Association analysis of the five ARGs and different clinical parameters in patients with HCC. The different clinical factors included pathologic stage **(A)** T stage, **(B)** M stage, **(C)** N stage, **(D)** gender, **(E)** age, **(F)** OS event, **(G)** and race **(H)**.

Delving into the potential clinical relevance of the five pivotal ARGs (BIRC5, E2F1, SFN, SPP1, and UBE2C), we examined how their expression levels interplay with various clinical parameters in HCC patients. Our investigative efforts unveiled critical associations, supported by significant correlations between the expression levels of these genes and key clinical features observed in HCC. Notably, we found substantial connections between the expression of BIRC5, E2F1, SFN, and UBE2C and the pathological stage of HCC (Figure 9A), indicating their potential as indicators of tumor progression and severity. Moreover, all five ARGs showed a strong association with both the extent of tumor invasion (T stage) and the overall survival of patients, further emphasizing their prognostic potential (Figures 9B, H). Additionally, the expression of UBE2C was found to be significantly related to the age of the patients (Figure 9E), suggesting that gene expression may vary across different age groups, potentially influencing prognosis. Correlations between BIRC5, E2F1, and UBE2C with patient race also emerged, indicating these ARGs as potential markers of biological variability across populations (Figure 9F). In contrast, no significant associations were observed between the five ARGs and the stage M, stage N, or gender in HCC patients (Figures 9C, D, G). This suggests that while the examined ARGs play a substantial role in predicting tumor stage and survival outcomes, they may not have as much influence on these other clinical characteristics. Overall, our findings highlight the importance of the T stage and overall survival as key determinants impacted by the ARGs, indicating that these genetic markers could be vital in understanding the trajectory of HCC progression and guiding personalized therapeutic strategies.

### 3.7 Establishment of ceRNA regulatory network

**FIGURE 10:**
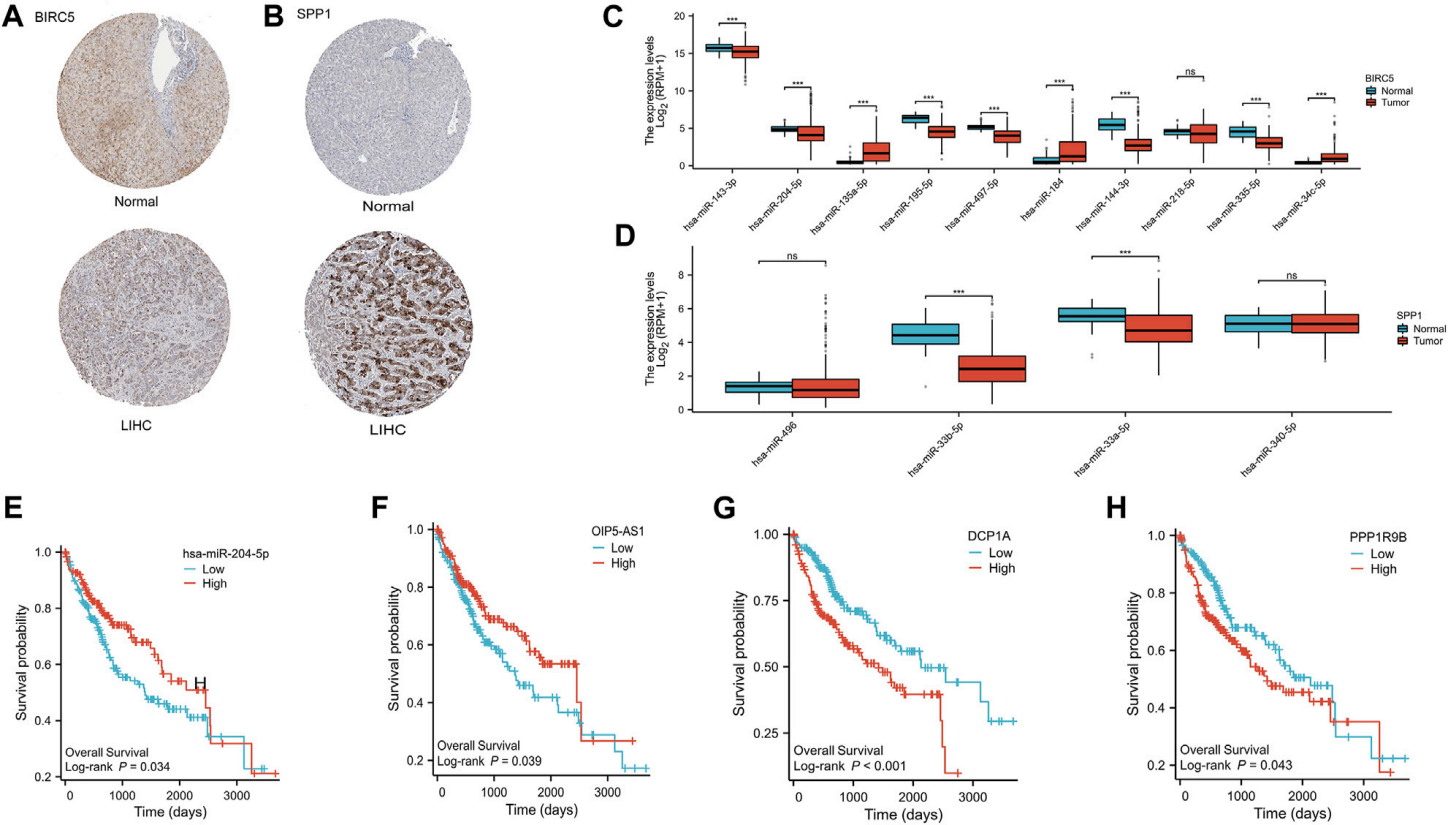
Construction of the ceRNA regulatory axis. **(A)** Differential expression of BIRC5 protein in HCC and normal liver tissues. **(B)** Differential expression of SPP1 protein in HCC and normal liver tissues. **(C)** Differential expression of the 10 miRNAs about BIRC5 in HCC and normal liver tissues. ns, *p* ≥ 0.05; *, *p* < 0.05; ***, *p* < 0.001. **(D)** Differential expression of the 4 miRNAs about SPP1 in HCC and normal liver tissues. ns, *p* ≥ 0.05; *, *p* < 0.05; ***, *p* < 0.001. **(E)** OS curves of has-miR-205-5p in patients with HCC in the low and high expression groups. **(F)** OS curves of OIP5-AS1 in patients with HCC in the low and high expression groups. **(G)** OS curves of DCP1A in patients with HCC in the low and high expression groups. **(H)** OS curves of PPP1R9B in patients with HCC in the low and high expression groups.

In our investigation of the functional significance of ARGs in HCC, we focused on two specific ARGs, BIRC5 and SPP1, to understand their roles in potential ceRNA networks that may influence HCC regulation. We examined the protein expression levels of BIRC5 and SPP1 using the HPA database, specifically utilizing antibodies HPA002830 and HPA027541, respectively. The dataset revealed a significant expression of these proteins in HCC patient samples (Figures 10A, B), confirming their clinical relevance. To identify the miRNA players within the ceRNA networks of BIRC5 and SPP1, we referred to the StarBase v3 database, which provided a list of potential miRNA targets (Supplementary Table S1). Through differential expression analysis of these miRNAs in HCC using the Wilcoxon rank-sum test, we identified significant variations. Regarding BIRC5, nine miRNAs showed significant modulation. This included upregulation of hsa-miR-143-3p, hsa-miR-204-5p, hsa-miR-195-5p, hsa-miR-497-5p, hsa-miR-144-3p, and hsa-miR-335-5p, while hsa-miR-135a-5p, hsa-miR-184, and hsa-miR-34c-5p were downregulated in HCC (Figure 10C). In SPP1, both hsa-miR-33b-5p and hsa-miR-33a-5p were found to be significantly downregulated in HCC (Figure 10D). Further analysis showed that elevated levels of hsa-miR-204-5p were associated with better survival outcomes for HCC patients (Figure 10E and Supplementary Figure S2), indicating its potential therapeutic value. By exploring the ceRNA network, the StarBase and ENCORI databases predicted 30 lncRNAs that could potentially interact with hsa-miR-204-5p (Supplementary Table S2). Among these, the expressions of OIP5-AS1, DCP1A, and PPP1R9B lncRNAs were found to be significantly associated with HCC prognosis using the log-rank test (Figures 10F–H and Supplementary Figure S4). Notably, high levels of DCP1A and PPP1R9B were linked to poorer survival rates in HCC patients (Figures 10G, H). Based on these findings, the ceRNA axes involving OIP5-AS1/hsa-miR-204-5p/BIRC5, DCP1A/hsa-miR-204-5p/BIRC5, and PPP1R9B/hsa-miR-204-5p/BIRC5 appear to play a crucial role in the progression of HCC, suggesting potential avenues for targeted therapeutic interventions in this type of cancer.

### 3.8 Screening of active ingredients for BIRC5 in TCM

In our investigation into the active ingredients that may affect the expression of the prognostically significant gene BIRC5 in HCC, we referred to the CTD to identify herbal compounds that could potentially interact with BIRC5. After analyzing the database, we found ten herbal compounds that could be potential candidates for further study: baicalein, berberine, curcumin, hyperoside, naringenin, platycodin D, quercetin, thymoquinone, resveratrol, and deguelin. The chemical structures of these compounds are listed in Table 2.

**TABLE 2:** The Molecular Docking result of active compounds related to BIRC5.

ID	Compound	Structure
1	Baicalein	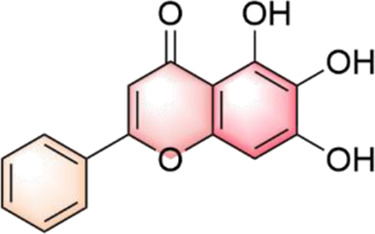
2	Berberine	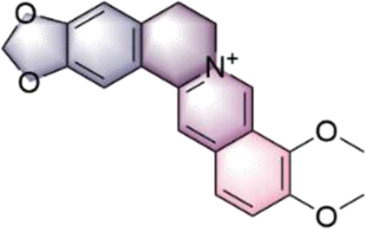
3	Curcumin	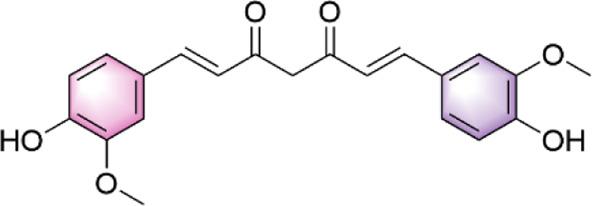
4	Hyperoside	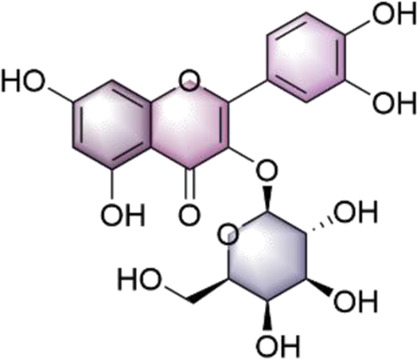
5	Naringenin	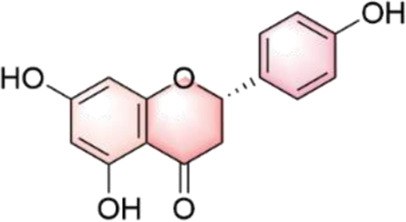
6	Platycodin D	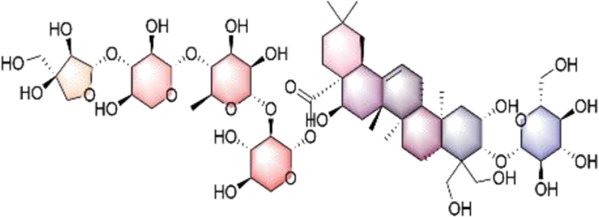
7	Quercetin	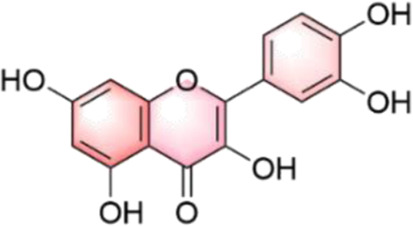
8	Thymoquinon	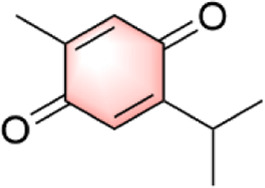
9	Resveratrol	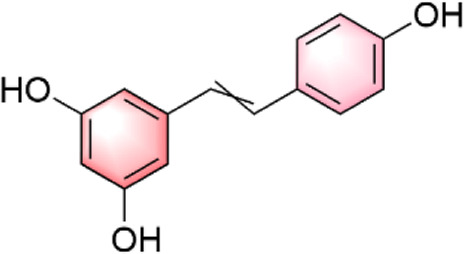
10	Deguelin	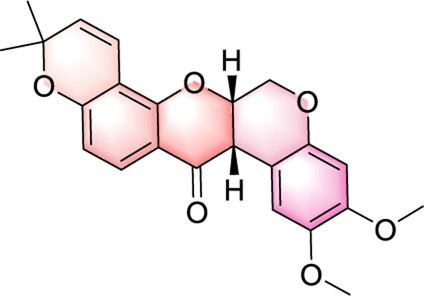

**FIGURE 11:**
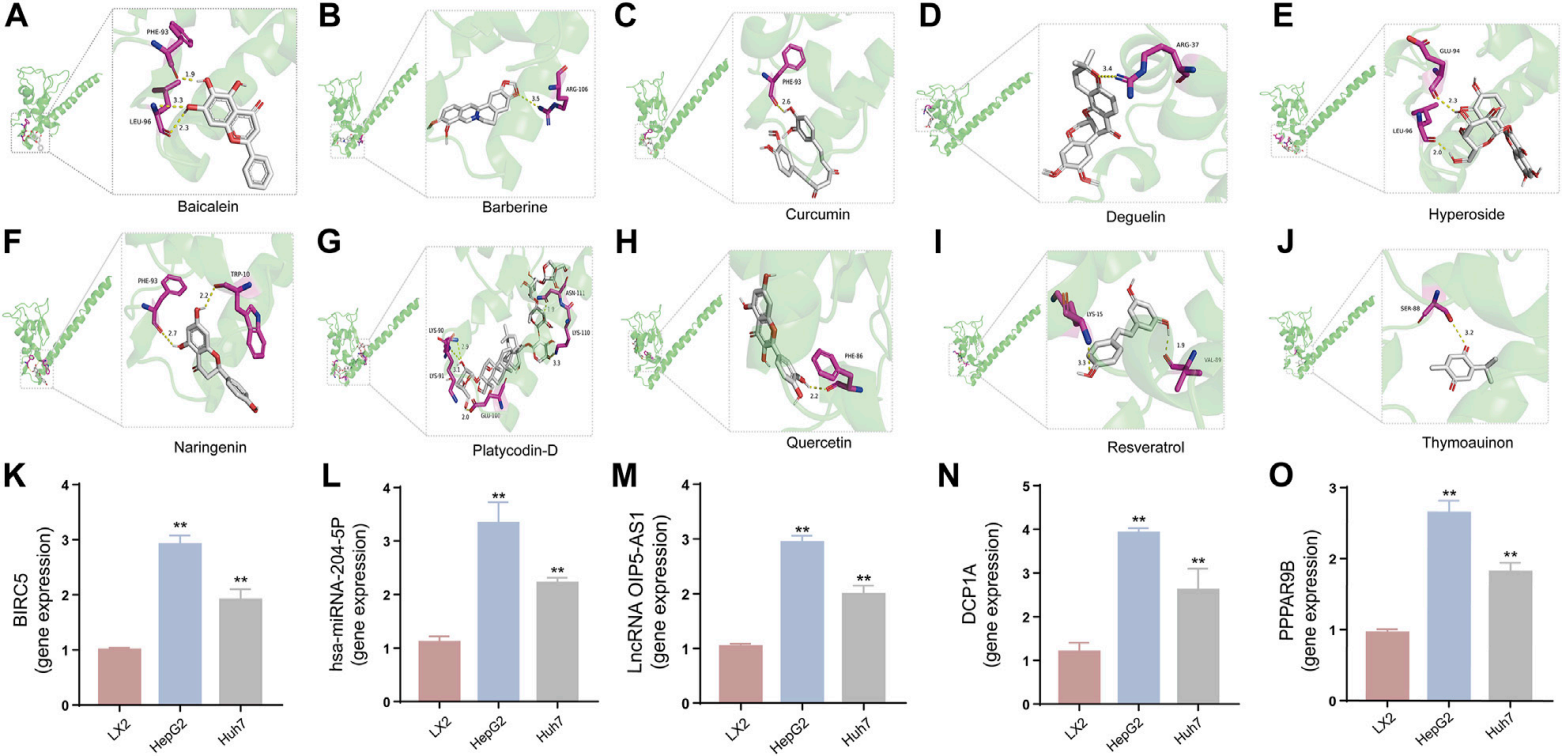
The interaction between ten TCM active components and BIRC5 protein was simulated by molecular docking **(A–J)** and the expression of the core genes in the regulatory network of three ceRNAs regulatory network in LX2, HepG2 and Huh7 cells based on qRT-PCR assay **(K–O)**. **(A)** Baicalein and BIRC5. **(B)** Berberine and BIRC5. **(C)** Curcumin and BIRC5. **(D)** Hyperoside and BIRC5. **(E)** Naringenin and BIRC5. **(F)** Platycodin D and BIRC5. **(G)** Quercetin and BIRC5. **(H)** Thymoquinon and BIRC5. **(I)** Resveratrol and BIRC5. **(J)** Deguelin and BIRC5. **(K)** BIRC5 gene expression. **(L)** hsa-miRNA-204-5p gene expression. **(M)** OIP5-AS1 gene expression. **(N)** DCP1A gene expression. **(O)** PPP1R9B gene expression. *p < 0.05, **p < 0.01, vs the LX2 group.

To refine our search for candidates with the most promising therapeutic potential, we utilized molecular docking techniques to evaluate the binding affinity between these phytochemicals and the target protein BIRC5. This methodological approach provided insights into the strength of the interactions between the compounds (ligands) and the receptor (BIRC5), as shown in Table 3. Typically, a ligand-receptor affinity score (binding energy) below −6.0 kcal/mol indicates strong binding and potential for functional interaction. Among the tested compounds, baicalin, platycodin D, and resveratrol demonstrated notable affinities for BIRC5, meeting the established criterion with significant ligand-receptor interactions. This suggests that these substances have the inherent ability to modulate the activity of BIRC5 (Figures 11A–J; Table 3). These findings provide a solid foundation for the hypothesis that these natural compounds could have meaningful implications in the treatment of HCC. Further *in vitro* and *in vivo* evaluations are necessary to validate their therapeutic efficacy and mechanism of action in relation to BIRC5.

**TABLE 3:** The binding ability of 10 active compounds related to BIRC5.

No	Compounds	Targets	PDB ID	Affinity (kcal/mol)
1	Baicalein	BIRC5	2qfa	−7.5
2	Berberine	BIRC5	2qfa	−7.2
3	Curcumin	BIRC5	2qfa	−6.5
4	Hyperoside	BIRC5	2qfa	−6.8
5	Naringenin	BIRC5	2qfa	−7.2
6	Platycodin D	BIRC5	2qfa	−7.5
7	Quercetin	BIRC5	2qfa	−7.2
8	Thymoquinon	BIRC5	2qfa	−6.8
9	Resveratrol	BIRC5	2qfa	−7.4
10	Deguelin	BIRC5	2qfa	−6.9

### 3.9 qRT-PCR and immunofluorescence staining analysis

**FIGURE 12:**
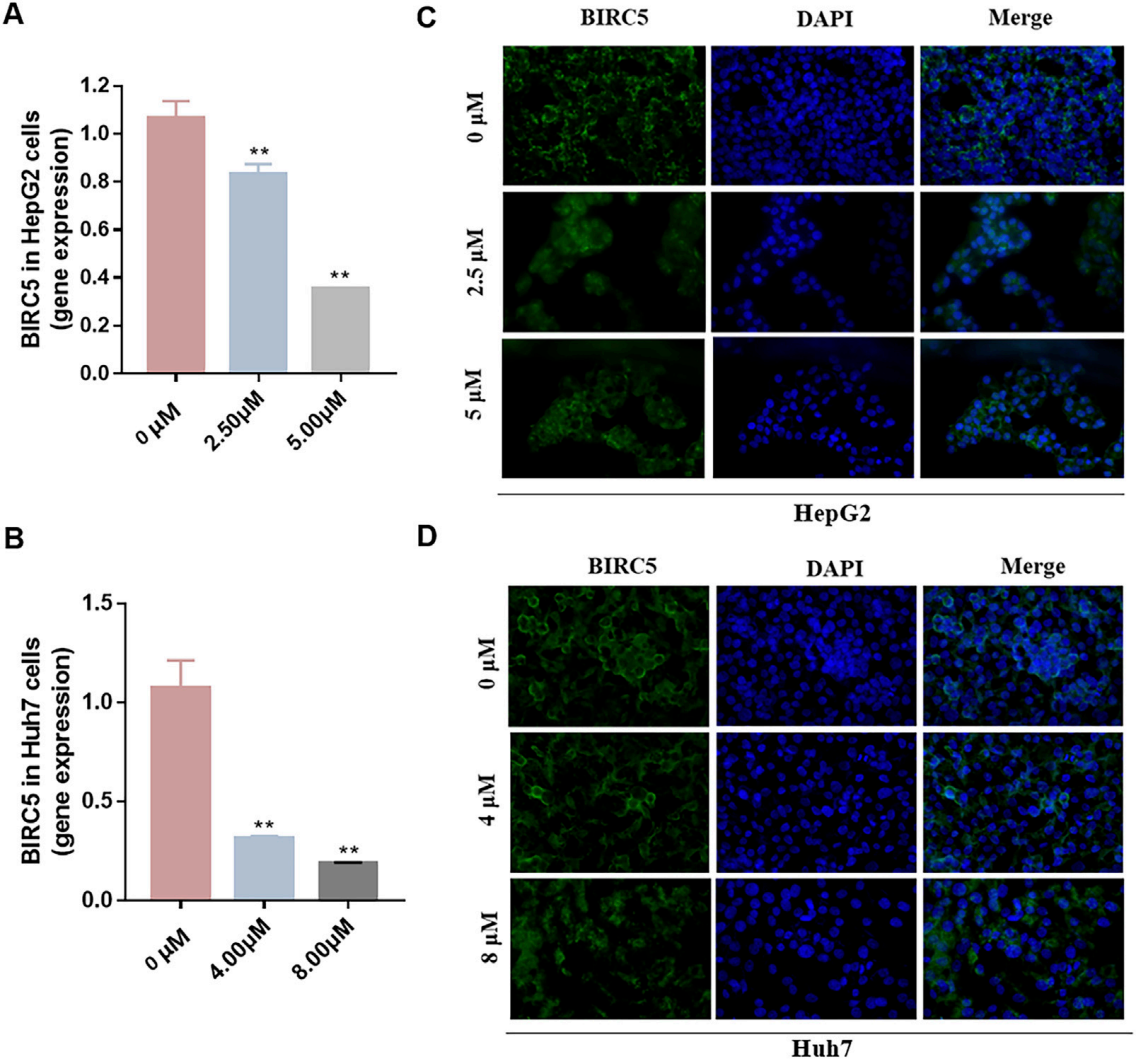
Effect of platycodin D on the expression of BIRC5 in HepG2 **(A)** cells and Huh7 **(B)** cells. The images of immunofluorescence staining in HepG2 **(C)** and Huh7 **(D)** cells treated with DMSO (control group) or platycodin D for 24 h. All images are taken with a field of view of 400x. *p < 0.05, **p < 0.01, vs the DMSO group.

To investigate the impact of three ceRNA networks on HCC progression, we conducted qRT-PCR assays. These assays were designed to validate the previously identified ceRNA regulatory networks within LX2, HepG2, and Huh-7. The results of these assays confirmed the accuracy and reliability of the predicted ceRNA interactions (Figures 11K–O), supporting our bioinformatic analyses and suggesting their involvement in the molecular etiology of HCC. Additionally, we explored the regulatory influence of platycodin D on the expression of our gene of interest, BIRC5, in HepG2 and Huh7 cell lines. The data obtained from these investigations further supported our *in silico* predictions, showing that platycodin D has a noticeable downregulatory effect on BIRC5 expression levels in both cell lines (Figures 12A, B, Supplementary Figure S4 and Supplementary Table S3). This observation is particularly relevant as it suggests a direct biological effect of platycodin D on a molecular target associated with HCC prognosis. To further investigate BIRC5’s response to platycodin D, we used immunofluorescence staining techniques to examine its subcellular localization and expression dynamics. Treatment of HepG2 and Huh7 cells with different doses of platycodin D resulted in a significant reduction in BIRC5 expression, providing further evidence of the compound’s ability to modulate this clinically relevant protein (Figures 12C, D). Overall, these findings highlight the therapeutic potential of platycodin D, particularly its ability to downregulate BIRC5, and suggest its potential as a novel therapeutic strategy in HCC treatment protocols.

## 4 Discussion

Anoikis is a protective mechanism that prevents detached cells from inappropriately adhering and proliferating, thereby preventing potential oncogenic transformation and the development of secondary tumors (Kakavandi et al., 2018). This type of cell death has been found to have significant prognostic and immunological implications in various cancers, such as lung adenocarcinoma (Diao et al., 2023), gastric cancer (Ye et al., 2020), and breast cancer (Li et al., 2019). Recent advancements in bioinformatics have led to groundbreaking discoveries in terms of therapeutic targets and drugs (Zhang et al., 2020; Cintron et al., 2023). However, there is still a lack of comprehensive bioinformatics characterization of ARGs in HCC, particularly in relation to immune infiltration, functional analysis, and predicting patient outcomes.

The present study identified 10 ARGs with significantly differential expression in HCC. Among these, seven ARGs (BIRC5, UBE2C, E2F1, NQO1, SFN, and SPINK1) were upregulated, while CXCL12, GDF2, and SLCO1B3 were downregulated, confirming their roles in HCC pathology. PPI networks are valuable in describing interrelationships between genes or proteins, including physical interactions and regulatory targeting, among others. They help elucidate meaningful molecular regulatory networks within organisms (Athanasios et al., 2017). Accordingly, through PPI analysis, we confirmed a strong association between BIRC5, UBE2C, E2F1, CXCL12, GDF2, NQO1, SFN, SPP1, and SPINK1. Furthermore, genetic mutations and alterations in molecular processes may promote HCC progression (Hashemi et al., 2023). Our mutation analysis suggested that GDF2 might have the highest mutation probability in HCC progression, followed by E2F1 and SLCO1B3. This finding is consistent with previous studies by Tan et al. and Sekine et al. (Sekine et al., 2011; Tan et al., 2022). The significant mutation probability of GDF2 could provide fresh insights for potential targeted interventions in HCC.

To investigate the roles and underlying mechanisms of ten newly identified ARGs, we conducted a functional enrichment analysis. Our KEGG pathway analysis revealed that these ARGs are mainly involved in various signal transduction pathways, such as the cell cycle, human cytomegalovirus infection, cytokine-receptor interactions, the PI3K/Akt signaling cascade, p53 signaling, and the Apelin signaling pathway. The cell cycle, which encompasses the G1, S, G2, and M phases, plays a crucial role in regulating cell proliferation (Yang et al., 2023). Disruptions in the cell cycle are characteristic features of cancer and have implications for various aspects of the disease, including metabolic processes, immunity, and metastatic potential (Cheung et al., 2023). Human cytomegalovirus, a member of the Herpesviridae family with a 236 kbp double-stranded DNA genome has been associated with dual outcomes (Dolan et al., 2004). Apart from causing illness in immunocompromised individuals, human cytomegalovirus is increasingly recognized for its involvement in tumor pathogenesis, as it has been detected in various tumor tissues such as glioblastoma and several adenocarcinomas (Pasquereau et al., 2017; Herbein, 2018; Nauclér et al., 2019). Emerging evidence suggests that human cytomegalovirus may contribute to advanced liver disease, providing valuable insights for the development of novel therapeutic approaches for HCC treatment (Cacicedo et al., 2022; Khalil et al., 2022). Cytokines, as crucial immune system mediators, interact with specific cellular receptors that are upregulated during cell activation in various diseases, particularly cancer (Scheller et al., 2021). The PI3K/Akt signaling pathway, which plays a vital role in cell growth, survival, metabolism, and angiogenesis, has been frequently found to be aberrantly activated in different types of malignancies (Hoxhaj and Manning, 2020). PTEN, a tumor suppressor that inhibits the PI3K/Akt pathway, has been extensively reported to counteract tumor growth in cancers of the endometrium, brain, skin, and prostate (Martini et al., 2014; Danielsen et al., 2015). Toll-like receptors (TLRs) are also implicated in tumor initiation and progression, particularly in cancers associated with chronic inflammation such as hepatic, colonic, gastric, and cervical carcinomas. Their role extends to promoting cell proliferation, inhibiting apoptosis, and facilitating immune evasion (Kaur et al., 2022). Furthermore, the tumor suppressor p53 functions as a key transcription regulator, initiating cell death signals and thereby inhibiting tumor development. For instance, Kandhavelu et al. demonstrated how alterations in the p53 signaling pathway influenced apoptosis and tumorigenesis in colon cancer (Kandhavelu et al., 2023). Despite the considerable knowledge of these signaling pathways in various cancers, research specifically investigating their significance and mechanisms in HCC remains limited. Further investigative efforts are warranted to gain a better understanding of their roles and therapeutic potential in HCC.

Using LASSO Cox regression analysis, we developed a prognostic model that incorporates five ARGs (BIRC5, E2F1, SFN, SPP1, and UBE2C), which have been found to be significant in the context of HCC. Previous studies have highlighted the prognostic importance of specific gene sets in HCC, including genes associated with ferroptosis (Zhang Y. et al., 2022; Zhao et al., 2022), glycolytic-related genes (Zou et al., 2022), and ARGs (Guizhen et al., 2022; Chen et al., 2023) are closely associated with HCC prognosis. Our analysis of the prognostic characteristic model for ARGs confirms its potential as a reliable prognostic tool for HCC patients, consistent with the findings of Chen et al., Guizhen et al., and Wang et al. (Wang et al., 2021; Guizhen et al., 2022; Chen et al., 2023). Among the selected genes, BIRC5 is an immune-related gene that inhibits apoptosis and promotes cell proliferation. There is increasing evidence suggesting the crucial role of BIRC5 in tumorigenesis, as it is overexpressed in various cancers such as breast cancer (Cao et al., 2023), oral squamous cell carcinoma (Cacına et al., 2023), benign meningioma (Maier et al., 2023), prostate cancer (Yu et al., 2023) and ovarian cancer (Li B. et al., 2023), thereby indicating a poor prognosis in cancer patients. Additionally, E2F1 is a well-known transcription factor that regulates the cell cycle and cell proliferation by binding to specific sites in the promoters of its downstream target genes to upregulate their expression. Overexpression of E2F1 during tumorigenesis can promote the malignant transformation of fibroblasts and induce liver carcinogenesis (Dong et al., 2023). Xiang et al. investigated the oncogenic and immunogenic effects of SPP1 in HCC and discovered that higher expression levels of SPP1 are associated with increased infiltration of immune cells. This finding is supported by previous studies on SFN (Li S. et al., 2023) and UBE2C (Shen et al., 2023), which have demonstrated significant impacts on cancer progression. Our bioinformatics analysis further supports these results. Overall, our study establishes a theoretical framework for future research on apoptosis-associated genes in the etiology and progression of HCC. Additionally, our prognostic assessment of ARGs in HCC provides a solid foundation for further extensive research in this field. Xiang et al. investigated the oncogenic and immunogenic effects of SPP1 in HCC and discovered that higher expression levels of SPP1 are associated with increased infiltration of immune cells (Xiang et al., 2023). This finding is supported by previous studies on SFN (Li S. et al., 2023) and UBE2C (Shen et al., 2023), which have demonstrated significant impacts on cancer progression. Our bioinformatics analysis further supports these results. Overall, our study establishes a theoretical framework for future research on apoptosis-associated genes in the etiology and progression of HCC. Additionally, our prognostic assessment of ARGs in HCC provides a solid foundation for further extensive research in this field.

Consistent with previous research, our investigation emphasizes the significant impact of interactions between tumor cells and immune infiltrates on the progression of cancer. The presence and behavior of infiltrating immune cells in the tumor microenvironment play a decisive role in both patient outcomes and the effectiveness of anti-neoplastic treatments (Shi et al., 2020). Building on these findings, our study reveals notable differences in the composition of infiltrating immune cell types between low-risk and high-risk groups in HCC, including macrophages, neutrophils, NK cells, mast cells, and activated memory CD4^+^ T cells. Activated macrophages often exhibit anti-tumor capabilities. In the context of HCC, M1 polarized macrophages can impede tumor growth through various pathways, while their M2 counterparts are involved in promoting angiogenesis, which facilitates tumor development (Cheng et al., 2022). Neutrophils have a dualistic role in oncogenesis, as they can either stimulate or inhibit cancer progression, highlighting their complex relationship with tumor pathobiology (Gryziak et al., 2022). NK cells are known for their innate cytotoxic potential and play a crucial role in lymphocyte-mediated effector responses. A reduced number of interferon-producing NK cells has been linked to advanced stages of HCC, suggesting their potential role in predicting disease prognosis (Lee et al., 2021). Furthermore, clinical observations have revealed a negative correlation between the presence of tryptase-positive mast cells and overall survival in HCC, indicating that mast cells could serve as a potential prognostic indicator for unfavorable clinical outcomes (Wang et al., 2018; Rohr-Udilova et al., 2021). Given these connections, it is crucial to thoroughly examine the function of immune cells in HCC pathology. These findings could provide valuable insights and have significant implications for the development of innovative therapeutic approaches that target the interaction between tumors and the immune system in HCC.

TCM has a long history of application in preventing and managing neoplasms, which has prompted intensive scientific inquiry into the discovery of TCM-derived anti-tumor agents. Our study aimed to investigate the phytochemical constituents of TCM for their potential modulatory effects on BIRC5, a gene implicated in tumor progression. Subsequent molecular docking confirmed the potential of three TCM ingredients, including baicalein, platycodin D, and resveratrol, as promising candidates. Resveratrol, a naturally occurring polyphenol, has garnered considerable attention for its versatile anti-neoplastic properties (Li et al., 2018). This compound is known to hinder the advancement of liver cancer by inhibiting the proliferation of precancerous cells and modulating apoptotic pathways. Specifically, it downregulates Bcl-2 and upregulates Bax expression in hepatocarcinogenesis (Bishayee and Dhir, 2009). Furthermore, resveratrol has been shown to attenuate oxidative stress, reduce pro-inflammatory cytokine levels, and initiate apoptosis by inhibiting SGK1 activity in the early stages of HCC development (Di Pascoli et al., 2013). Clinical investigations have demonstrated resveratrol’s ability to induce apoptosis in hepatic malignancies (Wang et al., 2023) and influence cell cycle regulators through the HGF/c-Met axis (Gao et al., 2017). Annaji et al. reported that resveratrol nanoparticles significantly enhance the anti-cancer potency against liver malignancies, both *in vitro* and *in vivo*, this positions resveratrol as a promising agent for liver cancer intervention (Annaji et al., 2021). Platycodin D, a triterpenoid saponin derived from *Platycodon grandiflorus*, has also demonstrated considerable anti-cancer efficacy against various malignancies, including HCC. Work by Hsu et al. illustrated that platycodin D may help overcome resistance to HDAC inhibitors in HCC by inhibiting the Erk1/2-regulated phosphorylation of CFL-1, offering a potential therapeutic approach to circumvent chemotherapy resistance (Hsu et al., 2021). Guo et al., noted that baicalin can inhibit the invasive and migratory capabilities of BEL-7402 cells by regulating cellular movement and the expression of MMP2, E-cadherin, TIMP2, and integrin β1. These findings collectively highlight the therapeutic potential of these herbal constituents in improving HCC prognosis (Guo, 2006). Collectively, these observations underscore the therapeutic potential of these herbal constituents in enhancing HCC prognosis. Our qRT-PCR and immunofluorescence assays investigating the expression of BIRC5 in HepG2 and Huh-7 cells support these observations. Therefore, our results indicate that platycodin D exhibits significant inhibitory action against HCC, providing a solid theoretical basis for further investigation.

In our investigation, we identified three potential ceRNA axes associated with HCC and discovered ten bioactive herbal components that have regulatory effects on BIRC5. Our molecular docking results were consistent with *in vitro* experiments, which further validated the predictions from our bioinformatics analyses. This comprehensive evaluation of HCC enhances our current understanding of its biological behaviors, clinical characteristics, and prognostic determinants, paving the way for more personalized and targeted therapeutic interventions. However, there are certain limitations in our research that need to be addressed in future studies. Firstly, the validation of our prognostic signature was limited to retrospective data obtained from the TCGA database. To establish its clinical relevance, prospective validations using diverse databases, including the GEO database, are essential. Secondly, the qRT-PCR based methods we used to validate RNA regulatory interactions have their own limitations, highlighting the need for more comprehensive evaluations involving both *in vitro* and *in vivo* models, as well as clinical trials. Furthermore, although our investigation suggested a connection between the prognostic model and immune infiltration, this potential link needs to be confirmed through studies with larger sample sizes. Further research is necessary to determine whether our model’s predictive capability remains strong when combined with immunotherapeutic approaches, and whether there are differential responses to such therapies between high-risk and low-risk patient groups. The impact of our prognostic signature on drug sensitivity also deserves thorough examination. A detailed analysis could provide valuable insights into how the gene signature in question modulates therapy resistance. Finally, the practice of studying Chinese medicines often revolves around the principle of “a single ingredient-a single medicine-a preparation”. This paradigm serves as a cornerstone of TCM research and represents a key area for our future investigative endeavors.

## 5 Conclusion

In summary, our analysis and experimental corroboration support the hypothesis that three competing endogenous RNA networks, namely, OIP5-AS1/hsa-miR-204-5p/BIRC5, DCP1A/hsa-miR-204-5p/BIRC5, and PPP1R9B/hsa-miR-204-5p/BIRC5, represent potential therapeutic intervention points for HCC. Additionally, our molecular docking studies identified baicalin, platycodin D, and resveratrol as potential modulators of BIRC5 expression in HCC, with platycodin D showing the most prominent effects. *In vitro* cellular experiments further validated these findings. Therefore, our study lays the groundwork for the development of precise pharmacological treatments for HCC. While our research offers promising avenues for HCC management, it also highlights the need for future investigations to refine and substantiate the therapeutic and prognostic insights obtained from our work.

## Data Availability

The datasets presented in this study can be found in online repositories. The names of the repository/repositories and accession number(s) can be found in the article/Supplementary Material.

## Abbreviations

Akt, Protein Kinase B; ARGs, Anoikis-related genes; BIRC5, Baculoviral IAP repeat-containing 5; CDK1, cyclin-dependent kinase 1; CXCL12, chemokine (C-X-C motif) ligand 12; DEGs, Differentially expressed genes; E2F1, Recombinant E2F transcription factor 1; GDF2, growth differentiation factor 2; GO, Gene ontology; GO-BP, GO biological processes; GO-CC, GO cell composition; GO-MF, GO molecular functions; HCC, Hepatocellular carcinoma; KEGG, Kyoto encyclopedia of genes and genomes; MSI, Microsatellite instability; NQO1, NADPH, quinone oxidoreductase1; OS, Overall survival; PI3K, Phosphoinositide 3-kinase; qRT-PCR, Quantitative real time polymerase chain reaction; SFN, Single frequency network; SGK1, Serum/glucocorticoid-inducible kinase 1; SLCO1B3, Solute carrier organic anion transporter family, member 1B3; SPINK1, Serine protease inhibitor kazal type 1; SPP1, Secreted phosphoprotein 1; TCM, Traditional Chinese medicine; TLRs, Toll-like receptor; TMB, Tumor mutation burden; UBE2C, Ubiquitin-conjugating enzyme E2C.

## References

[B1] Annaji M. Poudel I. Boddu S. H. S. Arnold R. D. Tiwari A. K. Babu R. J. (2021). Resveratrol-loaded nanomedicines for cancer applications. Cancer Rep. Hob. 4 (3), e1353. 10.1002/cnr2.1353 PMC8222557 33655717

[B2] Athanasios A. Charalampos V. Vasileios T. Ashraf G. M. (2017). Protein-protein interaction (PPI) network: recent advances in drug discovery. Curr. Drug Metab. 18 (1), 5–10. 10.2174/138920021801170119204832 28889796

[B3] Bishayee A. Dhir N. (2009). Resveratrol-mediated chemoprevention of diethylnitrosamine-initiated hepatocarcinogenesis: inhibition of cell proliferation and induction of apoptosis. Chem. Biol. Interact. 179 (2-3), 131–144. 10.1016/j.cbi.2008.11.015 19073162

[B4] Buchheit C. L. Angarola B. L. Steiner A. Weigel K. J. Schafer Z. T. (2015). Anoikis evasion in inflammatory breast cancer cells is mediated by Bim-EL sequestration. Cell Death Differ. 22 (8), 1275–1286. 10.1038/cdd.2014.209 25526094 PMC4495353

[B5] Cacicedo M. L. Limeres M. J. Gehring S. (2022). mRNA-based approaches to treating liver diseases. Cells 11 (20), 3328. 10.3390/cells11203328 36291194 PMC9601253

[B6] Cacına C. Akgün A. Kayhan K. B. Yaylım İ. Çakmakoğlu B. (2023). The analysis of Survivin promoter (-31G/C) gene variation in oral squamous cell carcinoma risk and prognosis. J. Stomatol. Oral Maxillofac. Surg. 124, 101494. 10.1016/j.jormas.2023.101494 37164127

[B7] Cao Q. Ai X. Q. Mushajiang M. (2023). Significance of nuclear factor-kappa B (NF-κB) and survivin in breast cancer and their association with radiosensitivity and prognosis. Breast Cancer (Dove Med. Press) 15, 175–188. 10.2147/bctt.S399994 36923396 PMC10010128

[B8] Chen Y. Huang W. Ouyang J. Wang J. Xie Z. (2023). Identification of anoikis-related subgroups and prognosis model in liver hepatocellular carcinoma. Int. J. Mol. Sci. 24 (3), 2862. 10.3390/ijms24032862 36769187 PMC9918018

[B9] Cheng K. Cai N. Zhu J. Yang X. Liang H. Zhang W. (2022). Tumor-associated macrophages in liver cancer: from mechanisms to therapy. Cancer Commun. (Lond) 42 (11), 1112–1140. 10.1002/cac2.12345 36069342 PMC9648394

[B10] Cheung A. H. Hui C. H. Wong K. Y. Liu X. Chen B. Kang W. (2023). Out of the cycle: impact of cell cycle aberrations on cancer metabolism and metastasis. Int. J. Cancer 152 (8), 1510–1525. 10.1002/ijc.34288 36093588

[B11] Cintron R. Whitmer S. L. M. Moscoso E. Campbell E. M. Kelly R. Talundzic E. (2023). HantaNet: a new MicrobeTrace application for hantavirus classification, genomic surveillance, epidemiology and outbreak investigations. Viruses 15 (11), 2208. 10.3390/v15112208 38005885 PMC10675615

[B12] D'Amato N. C. Rogers T. J. Gordon M. A. Greene L. I. Cochrane D. R. Spoelstra N. S. (2015). A TDO2-AhR signaling axis facilitates anoikis resistance and metastasis in triple-negative breast cancer. Cancer Res. 75 (21), 4651–4664. 10.1158/0008-5472.Can-15-2011 26363006 PMC4631670

[B13] Danielsen S. A. Eide P. W. Nesbakken A. Guren T. Leithe E. Lothe R. A. (2015). Portrait of the PI3K/AKT pathway in colorectal cancer. Biochim. Biophys. Acta 1855 (1), 104–121. 10.1016/j.bbcan.2014.09.008 25450577

[B14] Diao X. Guo C. Li S. (2023). Identification of a novel anoikis-related gene signature to predict prognosis and tumor microenvironment in lung adenocarcinoma. Thorac. Cancer 14 (3), 320–330. 10.1111/1759-7714.14766 36507553 PMC9870742

[B15] Di Pascoli M. Diví M. Rodríguez-Vilarrupla A. Rosado E. Gracia-Sancho J. Vilaseca M. (2013). Resveratrol improves intrahepatic endothelial dysfunction and reduces hepatic fibrosis and portal pressure in cirrhotic rats. J. Hepatol. 58 (5), 904–910. 10.1016/j.jhep.2012.12.012 23262250

[B16] Dolan A. Cunningham C. Hector R. D. Hassan-Walker A. F. Lee L. Addison C. (2004). Genetic content of wild-type human cytomegalovirus. J. Gen. Virol. 85 (5), 1301–1312. 10.1099/vir.0.79888-0 15105547

[B17] Dong R. Zhang D. Han B. Xu L. Zhang D. Cheng Z. (2023). DTL is a novel downstream gene of E2F1 that promotes the progression of hepatocellular carcinoma. Curr. Cancer Drug Targets 23, 817–828. 10.2174/1568009623666230511100246 37171007

[B18] Gao F. Deng G. Liu W. Zhou K. Li M. (2017). Resveratrol suppresses human hepatocellular carcinoma via targeting HGF-c-Met signaling pathway. Oncol. Rep. 37 (2), 1203–1211. 10.3892/or.2017.5347 28075467

[B19] Gryziak M. Wozniak K. Kraj L. Rog L. Stec R. (2022). The immune landscape of hepatocellular carcinoma-where we are? Oncol. Lett. 24 (5), 410. 10.3892/ol.2022.13530 36245826 PMC9555061

[B20] Guizhen Z. Weiwei Z. Yun W. Guangying C. Yize Z. Zujiang Y. (2022). An anoikis-based signature for predicting prognosis in hepatocellular carcinoma with machine learning. Front. Pharmacol. 13, 1096472. 10.3389/fphar.2022.1096472 36686684 PMC9846167

[B21] Guo Y. (2006). Experimental study on the effect and mechanism of baicalin on hepatocellular carcinoma cells *in vitro* . Shijiazhuang, China: Hebei Med Univ.

[B22] Han H. J. Sung J. Y. Kim S. H. Yun U. J. Kim H. Jang E. J. (2021). Fibronectin regulates anoikis resistance via cell aggregate formation. Cancer Lett. 508, 59–72. 10.1016/j.canlet.2021.03.011 33771684

[B23] Hashemi M. Nadafzadeh N. Imani M. H. Rajabi R. Ziaolhagh S. Bayanzadeh S. D. (2023). Targeting and regulation of autophagy in hepatocellular carcinoma: revisiting the molecular interactions and mechanisms for new therapy approaches. Cell Commun. Signal 21 (1), 32. 10.1186/s12964-023-01053-z 36759819 PMC9912665

[B24] Herbein G. (2018). The human cytomegalovirus, from oncomodulation to oncogenesis. Viruses 10 (8), 408. 10.3390/v10080408 30081496 PMC6115842

[B25] Hoxhaj G. Manning B. D. (2020). The PI3K-AKT network at the interface of oncogenic signalling and cancer metabolism. Nat. Rev. Cancer 20 (2), 74–88. 10.1038/s41568-019-0216-7 31686003 PMC7314312

[B26] Hsu W. C. Ramesh S. Shibu M. A. Chen M. C. Wang T. F. Day C. H. (2021). Platycodin D reverses histone deacetylase inhibitor resistance in hepatocellular carcinoma cells by repressing ERK1/2-mediated cofilin-1 phosphorylation. Phytomedicine 82, 153442. 10.1016/j.phymed.2020.153442 33412494

[B27] Huang D. Q. Mathurin P. Cortez-Pinto H. Loomba R. (2022). Global epidemiology of alcohol-associated cirrhosis and HCC: trends, projections and risk factors. Nat. Rev. Gastroenterology Hepatology 20 (1), 37–49. 10.1038/s41575-022-00688-6 36258033 PMC9579565

[B28] Ji A. Hu L. Ma D. Qiang G. Yan D. Zhang G. (2022). Myricetin induces apoptosis and protective autophagy through endoplasmic reticulum stress in hepatocellular carcinoma. Evid. Based Complement. Altern. Med. 2022, 3115312. 10.1155/2022/3115312 PMC9168098 35677365

[B29] Kakavandi E. Shahbahrami R. Goudarzi H. Eslami G. Faghihloo E. (2018). Anoikis resistance and oncoviruses. J. Cell Biochem. 119 (3), 2484–2491. 10.1002/jcb.26363 28836703

[B30] Kandhavelu J. Subramanian K. Naidoo V. Sebastianelli G. Doan P. Mani S. K. (2023). A novel EGFR Inhibitor, HNPMI regulates apoptosis and oncogenesis by modulating BCL-2/BAX and p53 in colon cancer. Br. J. Pharmacol. 181, 107–124. 10.1111/bph.16141 37183661 PMC10952184

[B31] Kaur A. Baldwin J. Brar D. Salunke D. B. Petrovsky N. (2022). Toll-like receptor (TLR) agonists as a driving force behind next-generation vaccine adjuvants and cancer therapeutics. Curr. Opin. Chem. Biol. 70, 102172. 10.1016/j.cbpa.2022.102172 35785601

[B32] Khalil F. O. Alsebaey A. Kasemy Z. A. Abdelmageed S. M. Bedair H. M. Abdelsattar S. (2022). IL28B, TLR7 SNPs, and cytomegalovirus infection are risk factors for advanced liver disease in chronic hepatitis C patients. Expert Rev. Anti Infect. Ther. 20 (1), 121–129. 10.1080/14787210.2021.1935239 34047252

[B33] Lee H. A. Goh H. G. Lee Y. S. Jung Y. K. Kim J. H. Yim H. J. (2021). Natural killer cell activity is a risk factor for the recurrence risk after curative treatment of hepatocellular carcinoma. BMC Gastroenterol. 21 (1), 258. 10.1186/s12876-021-01833-2 34118869 PMC8199695

[B34] Li B. Ding Z. Calbay O. Li Y. Li T. Jin L. (2023a). FAP is critical for ovarian cancer cell survival by sustaining NF-κB activation through recruitment of PRKDC in lipid rafts. Cancer Gene Ther. 30 (4), 608–621. 10.1038/s41417-022-00575-x 36494579 PMC10498436

[B35] Li S. Chen Y. Zhang Y. Jiang X. Jiang Y. Qin X. (2019). Shear stress promotes anoikis resistance of cancer cells via caveolin-1-dependent extrinsic and intrinsic apoptotic pathways. J. Cell Physiol. 234 (4), 3730–3743. 10.1002/jcp.27149 30171601

[B36] Li S. Wu H. Chen M. Tollefsbol T. O. (2023b). Combined broccoli sprouts and green tea polyphenols contribute to the prevention of estrogen receptor-negative mammary cancer via cell cycle arrest and inducing apoptosis in HER2/neu mice. J. Nutr. 151, 73–84. 10.1093/jn/nxaa315 PMC7779229 33188406

[B37] Li X. Li F. Wang F. Li J. Lin C. Du J. (2018). Resveratrol inhibits the proliferation of A549 cells by inhibiting the expression of COX-2. Onco Targets Ther. 11, 2981–2989. 10.2147/ott.S157613 29872310 PMC5973427

[B38] Maier A. D. Meddis A. Mirian C. Haslund-Vinding J. Bartek J. Krog S. M. (2023). Gene expression analysis during progression of malignant meningioma compared to benign meningioma. J. Neurosurg. 138 (5), 1302–1312. 10.3171/2022.7.Jns22585 36115056 PMC10353908

[B39] Martini M. De Santis M. C. Braccini L. Gulluni F. Hirsch E. (2014). PI3K/AKT signaling pathway and cancer: an updated review. Ann. Med. 46 (6), 372–383. 10.3109/07853890.2014.912836 24897931

[B40] Nauclér C. S. Geisler J. Vetvik K. (2019). The emerging role of human cytomegalovirus infection in human carcinogenesis: a review of current evidence and potential therapeutic implications. Oncotarget 10 (42), 4333–4347. 10.18632/oncotarget.27016 31303966 PMC6611507

[B41] Pasquereau S. Al Moussawi F. Karam W. Diab Assaf M. Kumar A. Herbein G. (2017). Cytomegalovirus, macrophages and breast cancer. Open Virol. J. 11, 15–27. 10.2174/1874357901711010015 28567162 PMC5420183

[B42] Rohr-Udilova N. Tsuchiya K. Timelthaler G. Salzmann M. Meischl T. Wöran K. (2021). Morphometric analysis of mast cells in tumor predicts recurrence of hepatocellular carcinoma after liver transplantation. Hepatol. Commun. 5 (11), 1939–1952. 10.1002/hep4.1770 34558826 PMC8557312

[B43] Rumgay H. Arnold M. Ferlay J. Lesi O. Cabasag C. J. Vignat J. (2022). Global burden of primary liver cancer in 2020 and predictions to 2040. J. Hepatol. 77 (6), 1598–1606. 10.1016/j.jhep.2022.08.021 36208844 PMC9670241

[B44] Scheller J. Berg A. Moll J. M. Floss D. M. Jungesblut C. (2021). Current status and relevance of single nucleotide polymorphisms in IL-6-/IL-12-type cytokine receptors. Cytokine 148, 155550. 10.1016/j.cyto.2021.155550 34217594

[B45] Sekine S. Ogawa R. Ojima H. Kanai Y. (2011). Expression of SLCO1B3 is associated with intratumoral cholestasis and CTNNB1 mutations in hepatocellular carcinoma. Cancer Sci. 102 (9), 1742–1747. 10.1111/j.1349-7006.2011.01990.x 21615622

[B46] Shen J. Yan H. Yang C. Lin H. Li F. Zhou J. (2023). Validation of a disease-free survival prediction model using UBE2C and clinical indicators in breast cancer patients. Breast Cancer (Dove Med. Press) 15, 295–310. 10.2147/bctt.S402109 37139241 PMC10149777

[B47] Shi J. Jiang D. Yang S. Zhang X. Wang J. Liu Y. (2020). LPAR1, correlated with immune infiltrates, is a potential prognostic biomarker in prostate cancer. Front. Oncol. 10, 846. 10.3389/fonc.2020.00846 32656075 PMC7325998

[B48] Sun Z. Zhao Y. Wei Y. Ding X. Tan C. Wang C. (2022). Identification and validation of an anoikis-associated gene signature to predict clinical character, stemness, IDH mutation, and immune filtration in glioblastoma. Front. Immunol. 13, 939523. 10.3389/fimmu.2022.939523 36091049 PMC9452727

[B49] Tan Z. Chen M. Peng F. Yang P. Peng Z. Zhang Z. (2022). E2F1 as a potential prognostic and therapeutic biomarker by affecting tumor development and immune microenvironment in hepatocellular carcinoma. Transl. Cancer Res. 11 (8), 2713–2732. 10.21037/tcr-22-218 36093522 PMC9459514

[B50] Wang L. Zhang W. Yang T. He L. Liao Y. Lu J. (2021). Construction and comprehensive analysis of a stratification system based on AGTRAP in patients with hepatocellular carcinoma. Dis. Markers 2021, 6144476. 10.1155/2021/6144476 34840632 PMC8612796

[B51] Wang N. Gao E. Cui C. Wang F. Ren H. Xu C. (2023). The combined anticancer of peanut skin procyanidins and resveratrol to CACO-2 colorectal cancer cells. Food Sci. Nutr. 11 (10), 6483–6497. 10.1002/fsn3.3590 37831732 PMC10563709

[B52] Wang S. Hu Y. Yan Y. Cheng Z. Liu T. (2018). Sotetsuflavone inhibits proliferation and induces apoptosis of A549 cells through ROS-mediated mitochondrial-dependent pathway. BMC Complement. Altern. Med. 18 (1), 235. 10.1186/s12906-018-2300-z 30092797 PMC6085663

[B53] Wang S. Xing N. Meng X. Xiang L. Zhang Y. (2022). Comprehensive bioinformatics analysis to identify a novel cuproptosis-related prognostic signature and its ceRNA regulatory axis and candidate traditional Chinese medicine active ingredients in lung adenocarcinoma. Front. Pharmacol. 13, 971867. 10.3389/fphar.2022.971867 36110528 PMC9468865

[B54] Xi S. Y. Minuk G. Y. (2018). Role of traditional Chinese medicine in the management of patients with hepatocellular carcinoma. World J. Hepatol. 10 (11), 799–806. 10.4254/wjh.v10.i11.799 30533181 PMC6280158

[B55] Xiang T. Cheng N. Huang B. Zhang X. Zeng P. (2023). Important oncogenic and immunogenic roles of SPP1 and CSF1 in hepatocellular carcinoma. Med. Oncol. 40 (6), 158. 10.1007/s12032-023-02024-7 37097499 PMC10129977

[B56] Yang R.-Y. Tan J.-Y. Liu Z. Shen X.-L. Hu Y.-J. (2023). Lappaol F regulates the cell cycle by activating CDKN1C/p57 in human colorectal cancer cells. Pharm. Biol. 61 (1), 337–344. 10.1080/13880209.2023.2172048 36708218 PMC9888477

[B57] Ye G. Yang Q. Lei X. Zhu X. Li F. He J. (2020). Nuclear MYH9-induced CTNNB1 transcription, targeted by staurosporin, promotes gastric cancer cell anoikis resistance and metastasis. Theranostics 10 (17), 7545–7560. 10.7150/thno.46001 32685004 PMC7359096

[B58] Yu Z. Chao H. Xu F. Deng H. Deng L. Song Z. (2023). Identification of a prognostic biomarker predicting biochemical recurrence and construction of a novel nomogram for prostate cancer. Front. Oncol. 13, 1115718. 10.3389/fonc.2023.1115718 37077837 PMC10106702

[B59] Zhang J. Shang L. Jiang W. Wu W. (2022a). Shikonin induces apoptosis and autophagy via downregulation of pyrroline-5-carboxylate reductase1 in hepatocellular carcinoma cells. Bioengineered 13 (3), 7904–7918. 10.1080/21655979.2022.2052673 35293266 PMC9208523

[B60] Zhang X. H. Zou Z. Q. Xu C. W. Shen Y. Z. Li D. (2011). Myricetin induces G2/M phase arrest in HepG2 cells by inhibiting the activity of the cyclin B/Cdc2 complex. Mol. Med. Rep. 4 (2), 273–277. 10.3892/mmr.2011.417 21468563

[B61] Zhang Y. Ren H. Zhang C. Li H. Guo Q. Xu H. (2022b). Development and validation of four ferroptosis-related gene signatures and their correlations with immune implication in hepatocellular carcinoma. Front. Immunol. 13, 1028054. 10.3389/fimmu.2022.1028054 36304446 PMC9592986

[B62] Zhang Z. Zhou L. Xie N. Nice E. C. Zhang T. Cui Y. (2020). Overcoming cancer therapeutic bottleneck by drug repurposing. Signal Transduct. Target Ther. 5 (1), 113. 10.1038/s41392-020-00213-8 32616710 PMC7331117

[B63] Zhao C. Zhang Z. Tao J. (2022). A novel ferroptosis-related signature for prediction of prognosis, immune profiles and drug sensitivity in hepatocellular carcinoma patients. Curr. Oncol. 29 (10), 6992–7011. 10.3390/curroncol29100550 36290827 PMC9601138

[B64] Zou J. Y. Huang Y. J. He J. Tang Z. X. Qin L. (2022). Glycolytic and fatty acid oxidation genes affect the treatment and prognosis of liver cancer. World J. Clin. Cases 10 (15), 4737–4760. 10.12998/wjcc.v10.i15.4737 35801051 PMC9198879

