# Supplementary figures and images for: Deciphering hepatocellular carcinoma pathogenesis and therapeutics: a study on anoikis, ceRNA regulatory network and traditional Chinese medicine
Source: Front Pharmacol. 2024 Jan 12;14:1325992. doi: 10.3389/fphar.2023.1325992 (PMC10811069; doi:10.3389/fphar.2023.1325992)

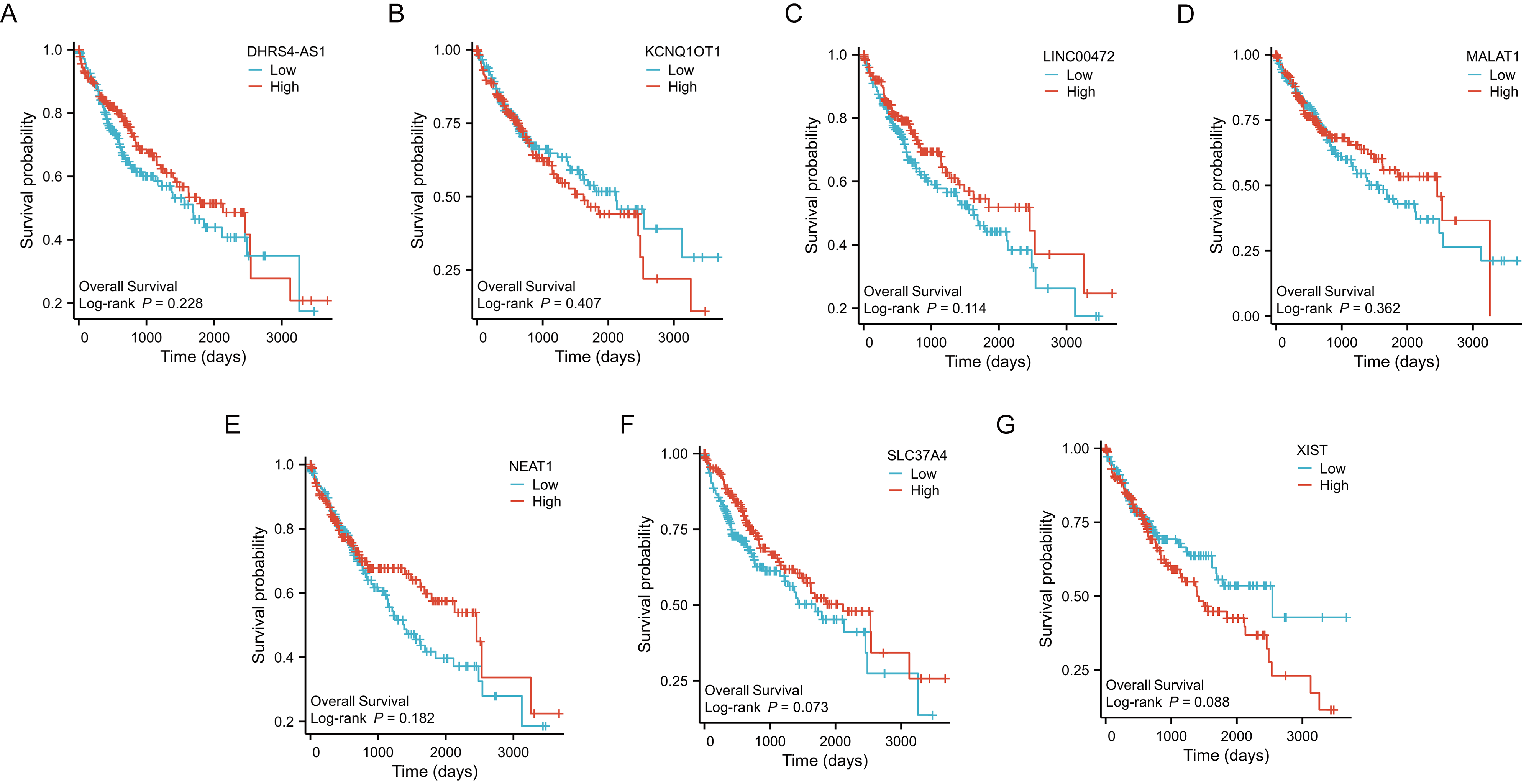

Supplement: Supplementary file 1 [file Image3.TIF]

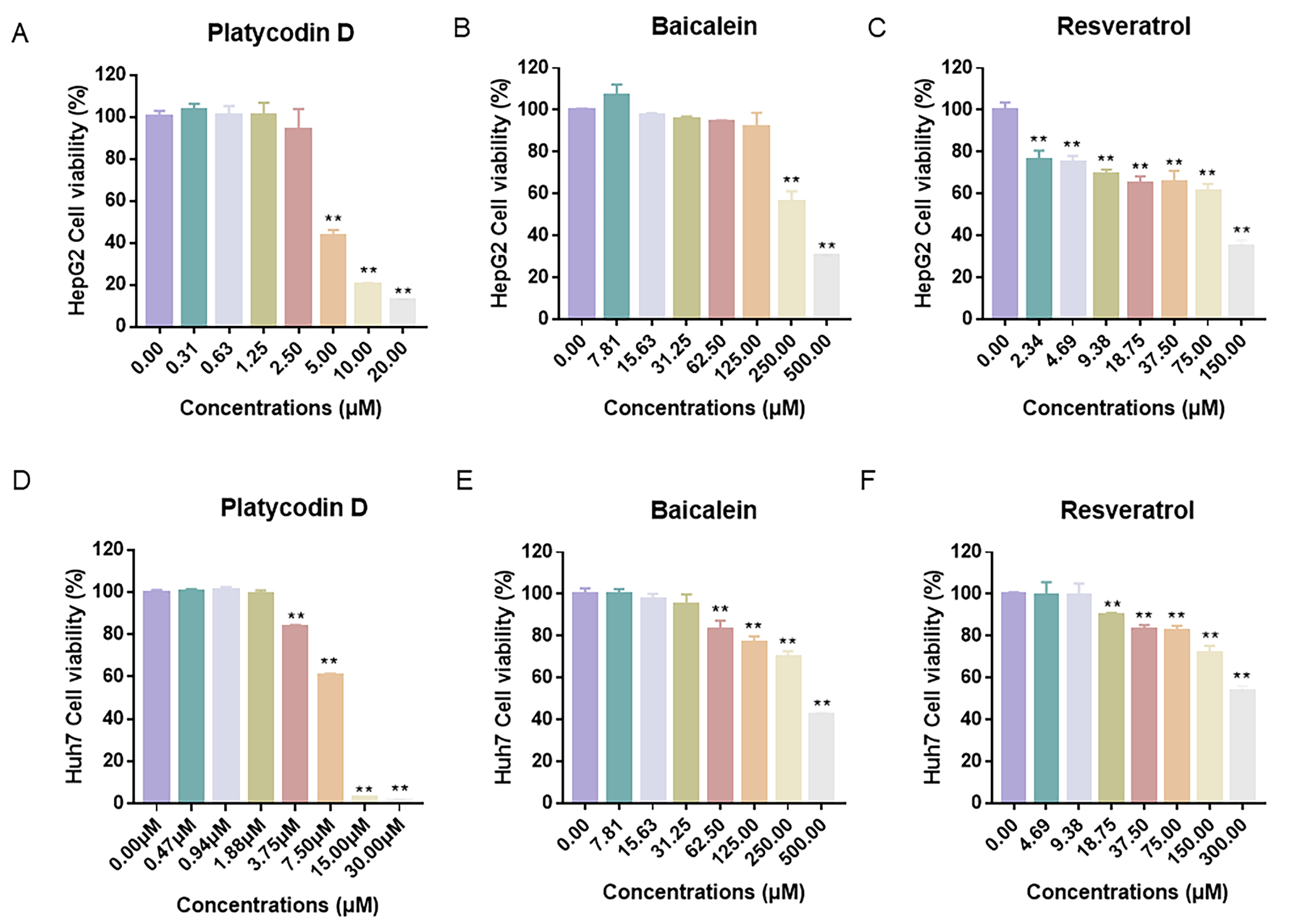

Supplement: Supplementary file 2 [file Image4.TIF]

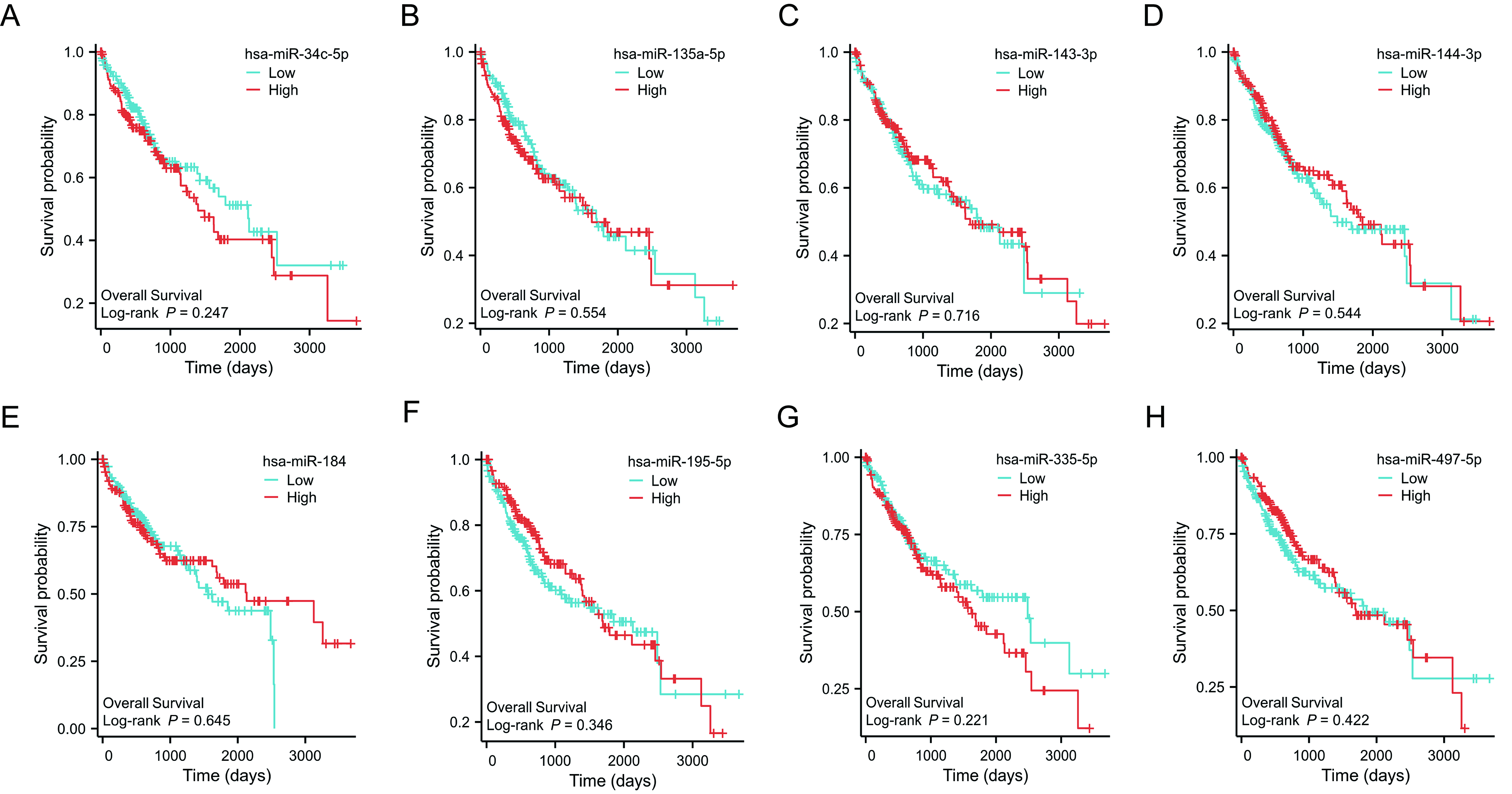

Supplement: Supplementary file 3 [file Image2.TIF]

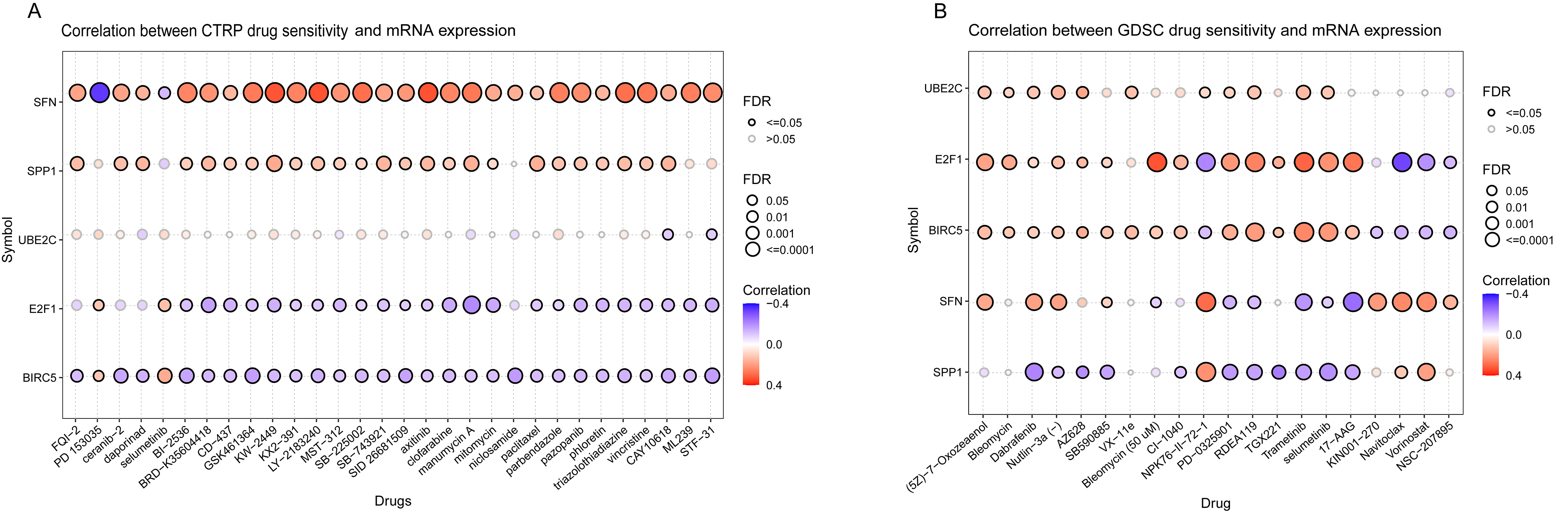

Supplement: Supplementary file 4 [file Image1.TIF]
