# Supplementary material for: Deciphering hepatocellular carcinoma pathogenesis and therapeutics: a study on anoikis, ceRNA regulatory network and traditional Chinese medicine
Source: Front Pharmacol. 2024 Jan 12;14:1325992. doi: 10.3389/fphar.2023.1325992 (PMC10811069; doi:10.3389/fphar.2023.1325992)
Supplement: Supplementary file 5 [file DataSheet1.docx]

**Supplementary materials**

**For**

**Deciphering hepatocellular carcinoma pathogenesis and therapeutics: A study on anoikis, ceRNA regulatory network and traditional Chinese medicine**

*Sa Guo^1^, Nan Xing^1^, Qinyun Du^1^, Bin Luo^2^, Shaohui Wang^3, 4, *^*

*^1^State Key Laboratory of Southwestern Chinese Medicine Resources, School of Pharmacy, Chengdu University of Traditional Chinese Medicine, Chengdu, 611137, China*

*^2^Shanghai Municipal Hospital of Traditional Chinese Medicine，Shanghai University of Traditional Chinese Medicine，Shanghai 200071, China*

*^3^State Key Laboratory of Southwestern Chinese Medicine Resources, School of Ethnic Medicine, Chengdu University of Traditional Chinese Medicine, Chengdu, 611137, China*

*^4^Meishan Hospital of Chengdu University of Traditional Chinese Medicine, Meishan, 620010, China*

**Corresponding authors:** Shaohui Wang (winter9091@163.com)

**Figure Legend for Supplementary Data**

**Supplementary Figure 1** Correlation between drug sensitivity and the 5 prognostic ARGs expression.

**Supplementary Figure 2** The OS curves of the 8 miRNAs in HCC and normal liver tissues.

**Supplementary Figure 3** The OS curves of 7 lncRNAs in HCC patients with low and high expression groups.

**Supplementary Figure 4** The CCK8 assay of Platycodin D, Baicalein and Reservatrol in HepG2 and Huh7 cells. *p < 0.05, **p < 0.01, vs. the DMSO group.

**Table Legend for Supplementary Data**

**Supplementary Table** **1** The information of the BIRC5 and SPP1 related miRNA.

**Supplementary Table** **2** The information of has-miR-204-5p related lncRNA.

**Supplementary Table** **3** The IC_50_ value of platycodin D, baicalin and resveratrol in HepG2 cells and Huh7 cells.

**Supplementary Figure 1** Correlation between drug sensitivity and the 5 prognostic ARGs expression.

**
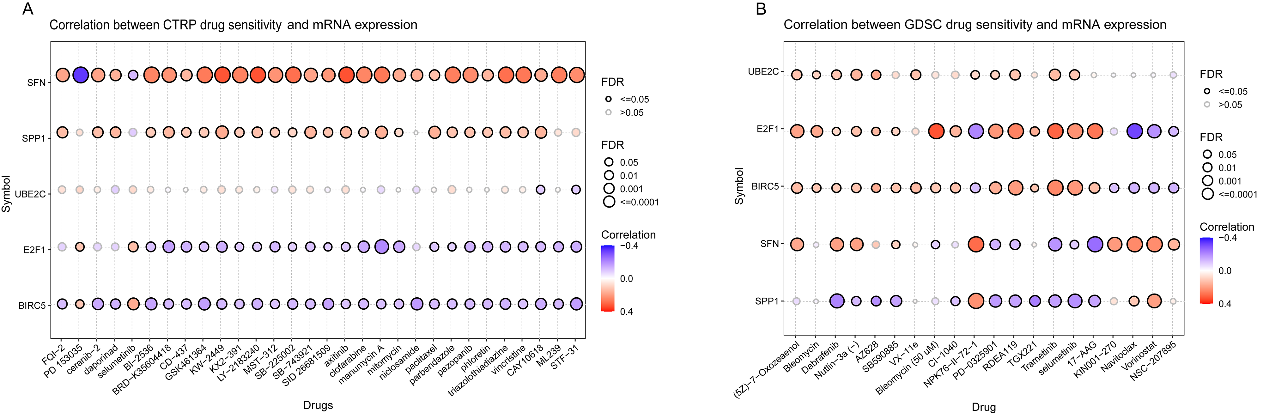
**

**Supplementary Figure 2** The OS curves of the 8 miRNAs in HCC and normal liver tissues.

**
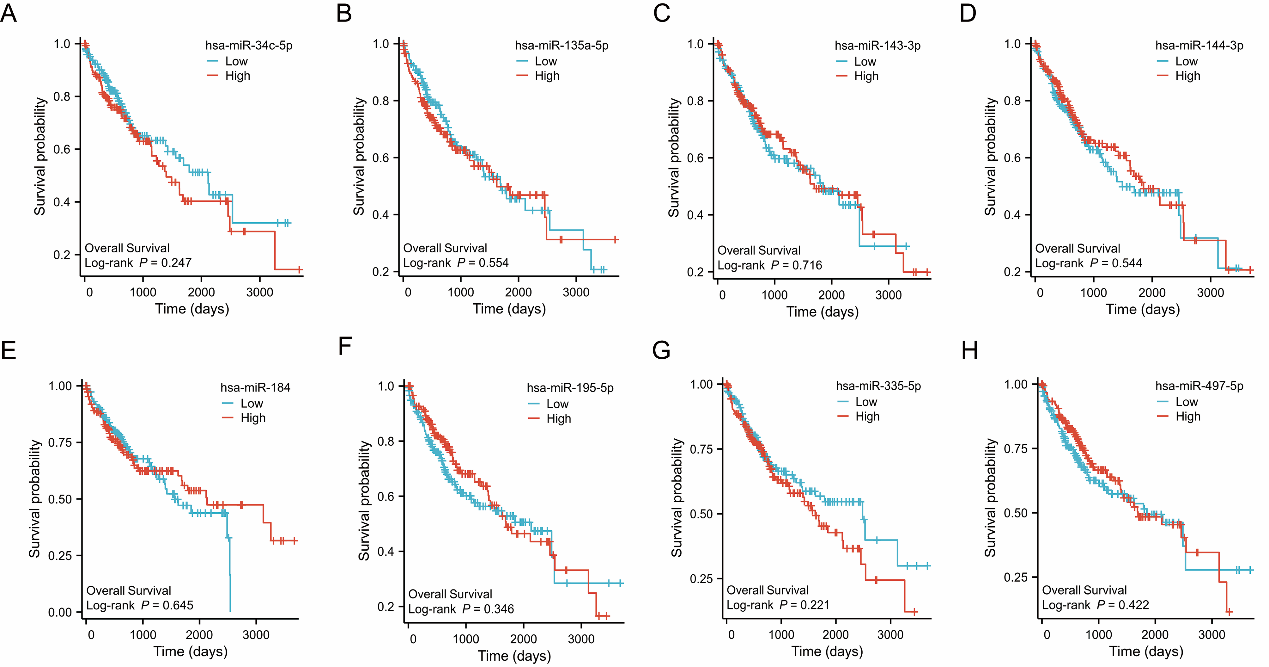
**

**Supplementary Figure 3** The OS curves of 7 lncRNAs in HCC patients with low and high expression groups.

**
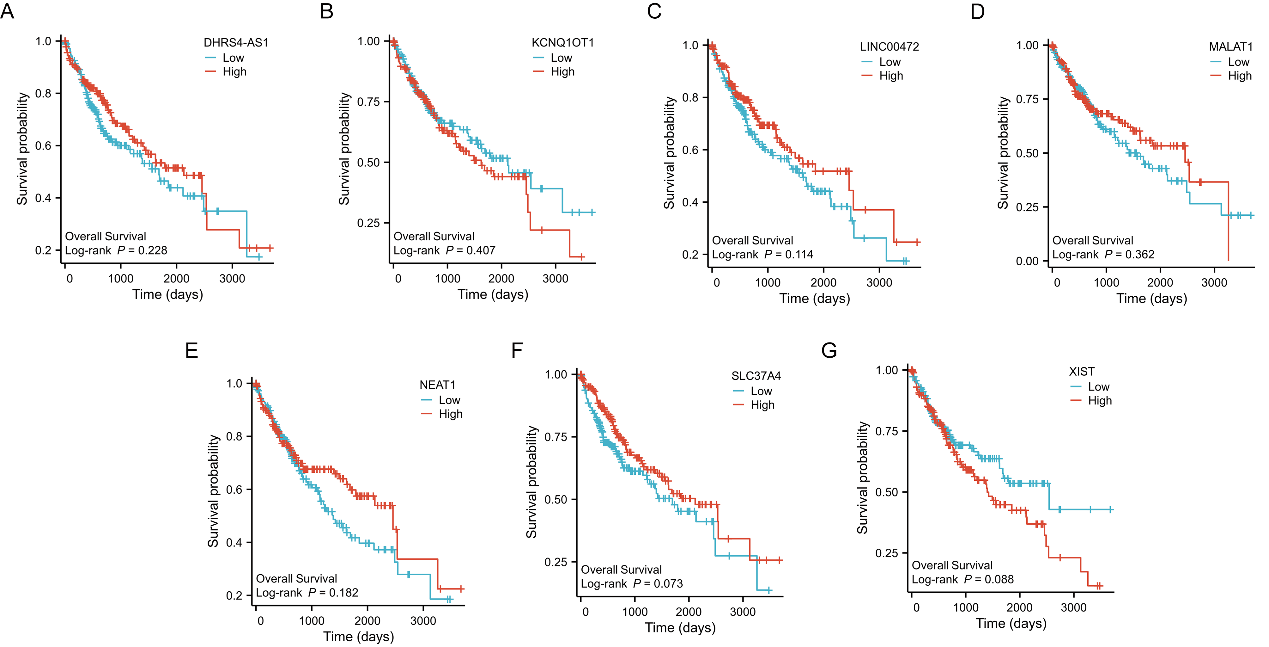
**

**Supplementary Figure 4** TheCCK8 assay of Platycodin D, Baicalein and Reservatrol in HepG2 and Huh7 cells. *p < 0.05, **p < 0.01, vs. the 0 μM group.

**
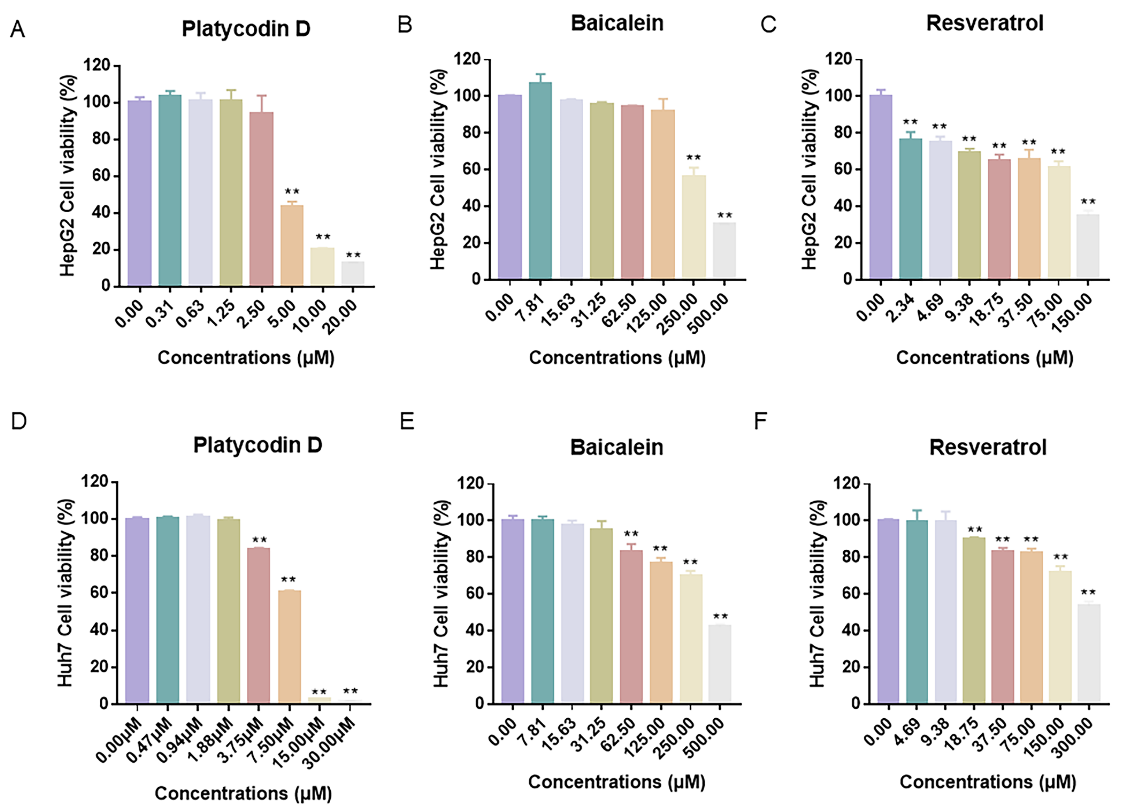
**

**Supplementary Table** **1** The information of the BIRC5 and SPP1 related miRNA.

| **Gene** | **miRNA** |
| --- | --- |
| BIRC5 | hsa-miR-143-3p, hsa-miR-204-5p, hsa-miR-135a-5p, hsa-miR-195-5p, hsa-miR-497-5p, hsa-miR-184, hsa-miR-144-3p, hsa-miR-218-5p, hsa-miR-335-5p, hsa-miR-34c-5p |
| SPP1 | hsa-miR-496, hsa-miR-33b-5p, hsa-miR-33a-5p, hsa-miR-340-5p |

**Supplementary Table** **2** The information of has-miR-204-5p related lncRNA.

| **miRNA** | **lncRNA** |
| --- | --- |
| has-miR-204-5p | RP11-258C19.5, HNRNPU-AS1, RP11-27M15.1, XIST, RP11-746E8.1, RP11-159D12.2, KCNQ1OT1, RP11-156E6.1, DCP1A, NEAT1, RP11-658F2.8, MCM3AP-AS1, RP11-220I1.1, SLC37A4, LINC00472, DHRS4-AS1, RP6-24A23.7, RP3-368A4.5, RP5-1024G6.8, CTA-204B4.6, CTB-96E2.3, RP1-37E16.12, RP3-331H24.5, RP4-773N10.5, RP11-435O5.2, CTC-281B15.1, MALAT1, PPP1R9B, RP11-252A24.7, OIP5-AS1 |

**Supplementary Table** **3** The IC_50_ value of platycodin D, baicalin and resveratrol in HepG2 cells and Huh7 cells.

|  | **HepG2 (μM)** | **Huh7 (μM)** |
| --- | --- | --- |
| Platycodin D | 5.00±0.13 | 8.00±0.03 |
| Baicalin | 309.67±4.56 | 423.20±3.19 |
| Resveratrol | 99.41±4.87 | 391.30±3.15 |
